# Longer Internode with Same Cell Length: *LcSOC1-b2* Gene Involved in Height to First Pod but Not Flowering in Lentil (*Lens culinaris* Medik.)

**DOI:** 10.3390/plants14081157

**Published:** 2025-04-08

**Authors:** Marzhan Kuzbakova, Gulmira Khassanova, Satyvaldy Jatayev, Nurgul Daniyeva, Crystal Sweetman, Colin L. D. Jenkins, Kathleen L. Soole, Yuri Shavrukov

**Affiliations:** 1 Faculty of Agronomy, S.Seifullin Kazakh AgroTechnical Research University, Astana 010000, Kazakhstan; khasanova-gulmira@mail.ru (G.K.); s.jatayev@kazatu.edu.kz (S.J.); 2 Core Facilities, Nazarbayev University, Astana 010000, Kazakhstan; nurgul.daniyeva@nu.edu.kz; 3 College of Science and Engineering, Biological Sciences, Flinders University, Adelaide, SA 5042, Australia; crystal.sweetman@flinders.edu.au (C.S.); colin.jenkins@flinders.edu.au (C.L.D.J.); kathleen.soole@flinders.edu.au (K.L.S.)

**Keywords:** cell length and width, epidermal cell, flowering time (FT), gene expression, haplotype, height to first pod (HFP), lentil, molecular marker ASQ, plant genotyping, scanning electron microscopy (SEM), *Suppressor of Overexpression of Constans 1* (*SOC1*) gene

## Abstract

Stem internode length determines height to first pod (HFP), an important trait for mechanical harvesting in legume crops. In the present study, this trait in lentil was (*Lens culinaris* Medik.) examined using scanning electron microscopy (SEM) of epidermal cells in stem internodes of two parents, Flip92-36L and ILL-1552, with long and short HFP, respectively. No significant differences in cell length, but differences in cell width were seen. This indicates that HFP was determined by cell number rather than cell length. The candidate gene family for HFP, *Suppressor of Overexpression of Constans 1* (*SOC1*), a member of the MADS-box transcription factor family, controls both flowering time (FT) and HFP traits. Six *LcSOC1* genes were identified in this study, and their expression was analysed. Most of the genes studied showed constitutive expression during vegetative growth, flowering, and seed development stages. Expression of *LcSOC1-a* seems to be involved in the transition to flowering and FT, whereas expression of *LcSOC1-b2* was strongly associated with HFP but not FT. Two haplotypes with two SNP each were identified in *LcSOC1-b2* among eight sequenced lentil accessions, and an SNP-based ASQ marker was developed and used for genotyping of a lentil germplasm collection. Significant association between *LcSOC1-b2* haplotypes and HFP was found in this study, indicating a primary role for this gene in internode length, potentially by regulating cell number.

## 1. Introduction

Height to first pod (HFP), defined as the distance from the soil level to the first pod in legume plants, is a very important trait for enabling mechanical harvesting [1]. HFP has increased during agricultural production, but selection of appropriate genotypes could provide a strong driver for improvement of this trait [2]. The genetic control of stem elongation and possible mechanisms resulting in HFP were recently reviewed in legumes [3]. In soybean (*Glycine max* L.), strong supporting evidence was reported for close associations between HFP and internode length [4]. Therefore, cell size, cell number or both could be directly involved in internode elongation and HFP.

The cell size in plant stems, including the epidermis, can vary across species and is influenced by many biological factors [5]. The elongation of cells is most often related to changes in gibberellic acid (GA) production or metabolism [6], as demonstrated in epidermal cells in stem internodes in overexpression lines of tobacco (*Nicotiana tabacum* L.) with *Arabidopsis* transgene [7], and in soybean hypocotyls [8], sensitive to GA.

Additionally, other plant hormones like auxins (AUX) can alter cell length, seen, for example, in the dramatic 10-fold shorter epidermal cells in the inflorescence of the AUX-defected *axr2* mutant of *Arabidopsis* [9]. Similarly, a mutant with the short internode gene *CmSi* in melon (*Cucumis melo* L.), interacting with AUX transporters genes, *ABCB* and *PIN-Formed*, showed significantly smaller stem cell area, particular in parenchyma compared to the wild-type (WT) [10]. However, a study on the *BR2* gene (*Brachytic2*), encoding AUX efflux P-glycoprotein-1 in maize (*Zea mays* L.), reported that cell length remained unchanged but cell numbers were reduced, resulting in shorter internodes [11]. For other hormones, like brassinosteroids (BS), *Arabidopsis* lines with overexpressed BS antagonist gene *Increased Leaf Inclination1 Binding,* and ectopic super compact 3, *scp-3*, in a dwarf mutant cucumber (*Cucumis sativus* L.), were reported to have shorter stem cells and smaller cell areas [12,13].

Less attention has been paid to stem cell width, with only a few studies which show effects of heavy metals; for example, significantly smaller width in epidermal cells in blueberry [14], and reduced epidermal thickness in stems of the lebbek tree, *Albizia lebbeck* [15]. Additionally, high light intensity can cause significant reduction of leaf epidermal cell thickness in orchid plants *Cypripedium macranthos* Sw. [16], whereas, the light spectrum was shown to influence changes in the cell size in *Astragalus membranaceus* Bunge plants [17]. However, a recent study with cluster bean [*Cyamopsis tetragonoloba* (L.) Taub.] suggested that width, but not length, of the cortex and xylem cells in the stem was reduced in response to drought and that this trait varied with drought tolerance, i.e., more tolerant lines demonstrated less stem width reduction [18].

In grapevine (*Vitis vinifera* L.), a short internode phenotype in the mutant Zijinzaosheng (ZJZS) was suggested to have reduced cell number rather than stem cell length with internodes shorter by about a half (47% of length) compared to WT cv. Venus Seedless [19]. There were no differences found for GA, AUX, cytokinins or abscisic acid in plants of the ZJZS mutant compared to WT. However, the authors reported that internode length was potentially regulated by jasmonic acid (JA). Based on RNAi results, the authors proposed that *TCP* genes (Teosinte branched 1/Cycloidea/Proliferating cell factors) are involved in this process. *TCP* transcription factors (TF), such as *AtTCP14* and *AtTCP15* in Arabidopsis, regulate internode cell growth, division and proliferation [20,21].

The *Suppressor of Overexpression of Constans 1* (*SOC1*) gene represents one group of the MADS-box TFs. They all contain the CArG-box motif in promoter regions which are bound by regulatory elements in response to light and signals from various hormones [22,23], including those described above. In general, *SOC1* primarily controls the transition of plants to flowering, and the flowering time (FT) [24] in various plant species, from pigeon pea [*Cajanus cajan* (L.) Huth] [25] to *Chrysanthemum morifolium* [23], and exotic bamboo (*Bambusa oldhamii*) [22]. Additionally, *SOC1* genes were shown to be sensitive to vernalisation in faba beans (*Vicia faba* L.) [26], interacting with *Vrn1* and regulating FT in knockout lines of bread wheat (*Triticum aestivum* L.) [27]. This gene was also involved in the control of plant height and enhanced seed yield in maize [28], seed development in soybean [29], as well as internode elongation and late flowering in plants with RNAi *OsMADS50* (=*OsSOC1*) in rice (*Oryza sativa* L.) [30].

Three groups of *SOC1* were described in *Medicago truncatula*, *SOC1-a*, *SOC1-b*, and *SOC1-c* [31,32], and this classification is currently used for other plant species with variable numbers of genes in each group. *MtSOC1-a* gene from the first group, was reported to control both FT and shorter internodes with smaller cell size in the mutant of *M. truncatula* compared to overexpressing transgenic plants and WT [32]. It was also obvious that epidermal cell length was affected in this study, whereas the width of the cells remained clearly unchanged.

Two other genes, *MtSOC1-b* and *MtSOC1-c*, were also proposed to be involved in control of FT and internode elongation [33]. However, *MtSOC1-b* could be redundant for FT because it was not affected in the mutant *Mtsoc1-b*, whereas all three genes of *MtSOC1-a*, *-b* and *-c*, could be cumulatively involved in the FT trait as shown in the triple mutant of *M. truncatula* [34]. Additionally, the *MtSOC1-c* gene was confirmed to be involved not only in plant flowering but also in embryo development, seed dormancy and primary stem elongation [32].

In lentil (*Lens culinaris* Medik.), only one gene *LcSOC1-b* was identified and described as controlling FT under different light quality (spectra) [35]. Comparison of plants grown under high and low ratio of red/far-red light showed that the expression of *LcSOC1-b* was up-regulated by 2–4-fold (log_2_ = 1.09–1.99) in lentil cv. Lupa and in *L. orientalis*, the wild progenitor of cultivated lentils, respectively. However, there was no reporting of internode length or HFP in this study [35].

Many techniques and methods are currently used for molecular studies of HFP and internode length, for example, Genotyping-by-Sequencing (GBS) in faba beans [36]. The GBS method is very suitable for mapping population analyses and the authors reported about 19 identified QTLs for HFP in faba beans [36]. However, in our study, we attempted SNP genotyping using our recently developed method of Allele-specific qPCR (ASQ) [37]. Based on the identified SNP in a proposed candidate gene, the genotyping must then be established and used for haplotype determination in parents and selected hybrid breeding lines following genotyping of the candidate *LcSOC1* in a lentil germplasm collection. The final assessment included genotyping and phenotyping of HFP and FT in the hybrids and the lentil germplasm collection. This is a classical association analysis method used quite commonly for the verification of identified genes and studied traits [38,39].

In the present study, we aimed to find candidate genes controlling stem cell size, internode length and HFP in lentil. A candidate gene was identified during the analysis of the entire family of *LcSOC1,* through gene expression in lentil accessions with contrasting long and short internodes and HFP, using RT-qPCR, which is now a routine method [40,41]. Changes in gene expression (up- or down-regulation) during plant development in genotypes with differing HFP were associated with the trait. Additionally, to provide stronger validation of the identified candidate gene, it was important to study the gene expression not only in parents of lentil hybrid populations but also in advanced hybrid breeding lines, as conducted previously, for example, in tomato (*Solanum lycopersicum* L.) [42] and chickpea (*Cicer arietinum* L.) [43].

To study the role of *LcSOC1* genes in HFP, the following approaches were used: (1) Scanning electron microscopy analyses of epidermal cell size in stem internodes of two lentil accessions, Flip92-36L and ILL-1552, with contrasting long and short HFP; (2) *LcSOC1* candidate gene family global expression analysis using qRT-PCR; (3) SNP identification in *LcSOC1-b2* gene as the most suitable candidate gene for the HFP trait in lentil; (4) Plant genotyping for *LcSOC1-b2* in parents, hybrid breeding lines and in the lentil germplasm collection for their association with HFP.

## 2. Materials and Methods

### 2.1. Plant Material and Trait Evaluations

Seeds of lentil (*Lens culinaris* Medik.) accessions were provided by S.Seifullin Kazakh AgroTechnical Research University (KATRU), Astana (Kazakhstan), Vavilov Research Institute of Plant Genetic Resources (VRIPGR), St. Petersburg (Russia) [44] and from a local breeding collection.

The full list of the 66 lentil germplasms studied is presented in the Supplementary Materials Table S1. It includes eight lentil accessions selected for Sanger sequencing, four of which were used for hybridisations in previous steps. Three of the parents originated from the ICARDA germplasm collection (Syria): Flip92-36L, Flip96-48L, and ILL-1552, whereas the fourth one was a local cv. Krapinka from Kazakhstan [45]. The choice of the lentil accessions was based on initial observation and scores during preliminary experiments in the hybridisation program.

Two hybrid populations were established (1) ♀Flip92-36L × ♂ILL-1552, and (2) ♀Krapinka × ♂Flip96-48L. Originally, 10 F_2_ breeding lines were developed in each population, and three breeding lines from each population were selected for qPCR analysis.

For microscopy and molecular experiments with parents and hybrids, seeds were sown in 20 cm diameter pots with 3.0 kg of soil-mix with equal volumes of commercial potting mix and soil from a research field nearby KATRU (Kazakhstan). Plants were grown at KATRU Campus during the summer season with monitoring temperature and light intensity using Field Scout 3415F light meter (Spectrum Technology, Aurora, IL, USA) showing typical climate conditions as in research fields. Pots were watered twice weekly on a portable scale, keeping soil moisture level consistent at 80% field capacity.

For the lentil germplasm collection analysis, plants were grown in the research field over three years (2021–2023), from where soil was taken for pots, as described above. Each genotype was grown in a single row, 4 m in length, 10 cm between plants in row, and 60 cm between rows with three fully randomised replicates.

**Figure 1:**
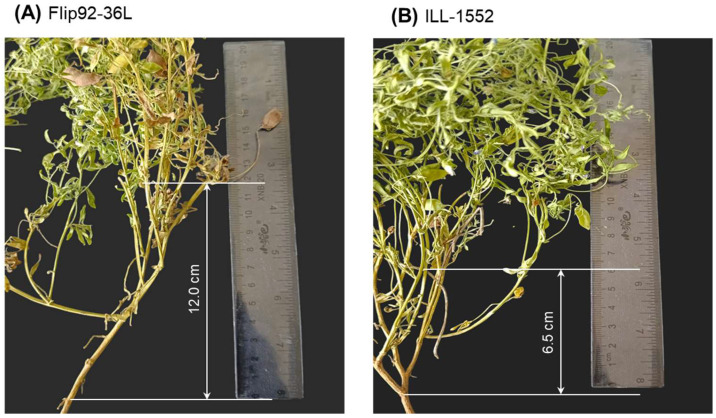
Images of lentil plants of accessions Flip92-36L (**A**) and ILL-1552 (**B**) with indicated HFP measurements.

Two major traits were evaluated in the current study: (1) height to first pod (HFP), measured as the distance from the soil surface to the first pods, using a centimetre ruler; and (2) flowering time (FT) as the number of days recorded from seed sowing until the first three flowers opened. An example of HFP measurement in two lentil accession is presented in Figure 1.

### 2.2. Scanning Electron Microscopy of Stem Epidepmal Cells

Lentil plants of two accessions, Flip92-36L and ILL-1552, were grown in pots with soil as described above for three weeks post germination. Stem samples were collected from the middle of the stem portion between roots and the first lateral branch from three plants, each representing a biological replicate, in plastic tubes. In the laboratory, three fragments about 5 mm in length were gently cut from each stem sample targeting internodes, with further sample preparation as described earlier [46,47] with some modifications.

Briefly, internode samples were fixed in 2.5% glutaraldehyde with 0.1 M phosphate buffer (pH 7.4) for 24 h and washed for 10 min, three times, with the same phosphate buffer. For the post-fixation step, samples were treated with 1% OsO_4_ in phosphate buffer for 2 h and washed for 10 min twice with phosphate buffer. Samples were dehydrated following the adjusted protocol [48], where two ethanol grade series were used, 30% and 50%, twice for 10 min each, and 70%, 80% and 96%, twice for 15 min for each change in ethanol solution. Critical point drying (Model K850, Quorum Technologies, Laughton, UK) in liquid carbon dioxide was used to remove any remaining solvent and to prevent sample surface damage. The dried internode samples were mounted on stubs and coated with a layer of 15 nm gold [49,50].

The epidermal surface of the samples was studied with a Scanning electron microscope (SEM), FIB-Auriga Crossbeam 540 (Carl Zeiss, Oberkochen, Germany) at an accelerating voltage of 5 kV with supplementary InLens detector. The images were produced using ×100 or ×200 magnification [51], and at least two best images with clear epidermis cells were selected for each biological replicate, with six images per genotype. These analyses were carried out in the Electron microscopy laboratory, Core Facilities and HPC, Nazarbayev University, Astana (Kazakhstan).

### 2.3. Identification of LcSOC1 Gene in Lentil

The sequences of three *SOC1* genes in *M. truncatula* were retrieved from the database of GenomeNet (Kyoto University, Japan) [52], using *MtSOC1-a* (Medtr7g075870), *MtSOC1-b* (Medtr8g033250) and *MtSOC1-c* (Medtr8g033220), as in the published report [32]. These *MtSOC1* sequences were used to search the same database of homologs in lentil, soybean and chickpea, as closely related legumes. Chromosome locations, positions on the physical map and *LcSOC1* gene identifications in lentil were based on database PCD (Pulse Crops Database) [53], whereas those for *GmSOC1* in soybean and *CaSOC1* in chickpea were found in database LIS (Legume Information System) [54]. All sequences of the identified *SOC1* genes and encoded proteins in the studied legume species were downloaded from GenomeNet, PCD, and LIS databases for molecular phylogenetic analysis and are presented in Supplementary Materials Table S2. The molecular-phylogenetic dendrogram was constructed using CLUSTALW Multiple Sequence Alignment at GenomeNet Database Resources [52]. The results file was converted into a ‘.nex’ file for further use in SplitsTree4, version 4.14.4, from algorithms in the bioinformatics website at the University of Tübingen, Germany [55].

### 2.4. RNA Extraction, cDNA Synthesis and qPCR Gene Expression Analysis

Leaf samples were collected from lentil plants at three stages: (1) three week old plants designated as ‘vegetative’ or ‘before flowering’; (2) plants carrying their first three flowers, designated as ‘flowering’; and (3) three weeks after the initiation of flowering, regarded as ‘seed development’. Three biological replicates from each genotype of parents and hybrid lines described in Section 2.2 were randomly selected and leaves snap-frozen in liquid nitrogen and stored at −80 °C. Leaf samples were ground with two 8-mm stainless ball bearings using a Vortex mixer, keeping the samples frozen. TRIzol-like reagent was used for RNA extraction following a previously described protocol [56]. Each sample, using 2 μg of RNA, was reverse transcribed using ОТ-M-MuLV-RH Reverse Transcriptase kit with supplementary DNase treatment (Biolab-Mix, Novosibirsk, Russia).

Samples of cDNA diluted with water (1:10) were used for qPCR analyses with a QuantStudio-7 Real-Time PCR system instrument (Thermo Fisher Scientific, Waltham, MA, USA), as mentioned in Section 2.6, following the qPCR protocol described earlier [57] with some modifications. The total volume (10 μL) of qPCR in each well included 5 μL of 2×Biomaster HS-qPCR SYBR Blue (Biolab-Mix, Novosibirsk, Russia), 4 μL of diluted cDNA, and 1 μL of two gene-specific primers (3 μM of each primer) as per the manufacturer’s recommendation (Supplementary Materials Table S3).

Thermal cycling conditions included a brief initial melt at 95 °C for 3 min, followed by 40 cycles of 95 °C for 5 s and 60 °C for 20 s, and finished with a melt curve from 60 °C to 95 °C increasing by 0.5 °C increments every 5 s. The efficiencies of all qPCR primers were calculated based on the slope of the corresponding calibration line, and their suitability was confirmed. Specificities of target and reference genes amplifications were verified with single distinct peaks on a melting curve and a single band of the expected size in a 2% agarose gel.

Expression data for the target genes were calculated with the normalisation of gene expression relative to geometric average expression [58] of the two reference genes: *LcELF4A-III*, Eukaryotic initiation factor 4A-III (Lcu.2RBY.2g019770), based on orthology with At3g13920 in *Arabidopsis thaliana* [59], and *LcActin-7* (Lcu.2RBY.L011470) [35], and their sequences and amplicon sizes are presented in Supplementary Materials Table S3. At least three biological and two technical replicates were used for each sample in qPCR experiments.

The Relative Standard Dilution method was used based on the ABI Guide for relative quantitation of gene expression using real-time quantitative PCR [60], where serial dilutions were applied for each target and reference gene individually. Threshold cycle values were determined based on linear calibration of template cDNA dilution factor and Cq value.

### 2.5. DNA Extraction, PCR, Sequencing and SNP Identification

Two to three young leaf samples were collected in 10-mL tubes from individual plants and frozen in liquid nitrogen. DNA was extracted using the CTAB-method as described earlier [61] with minor modifications. Frozen leaf samples were ground with ball bearings and Vortex-mixer keeping samples frozen, as described for RNA extraction in Section 2.4. The washed and dried DNA pellet was finally dissolved in 100 µL of 1/10 diluted TE Buffer with 25 µg of RNase A added. The DNA concentration was measured by Nano-Drop spectrophotometer (Thermo Fisher Scientific, Waltham, MA, USA), and the DNA quality was assessed on a 1% agarose gel.

The primers were designed based on the DNA sequence of *LcSOC1-b2* gene designated as Lcu.2RBY.7g014090, from the *L. culinaris* reference genome of cv. Redberry, targeting two genetic fragments in the first intron, 1081 and 891 bp, respectively. The sequences of the primers as well as the entire Lcu.2RBY.7g014090 gene are present in Supplementary Materials Table S4. Regular PCR conditions as described earlier [57] were used, with some modifications for sequencing. Briefly, the total reaction volume was increased to 60 μL and contained 6 μL of template lentil DNA (20 ng/mL) with the following components in their final concentrations for each reaction: 1 × SE PCR buffer including 1.5 mM MgCl_2_, 0.2 mM of each dNTPs, 0.25 mM of each primer, and 1.0 U of E332 Taq DNA polymerase (SibEnzyme, Novosibirsk, Russia). PCR was conducted on a SimpliAmp Cycler (Thermo Fisher Scientific, Waltham, MA, USA) using a program with the following steps: initial denaturation, 94 °C for 2 min; 35 cycles of 94 °C for 15 s, 55 °C for 15 s, and 72 °C for 1 min, and a final extension of 72 °C for 3 min.

The PCR products were purified using a PCR Purification kit (Syntol, Moscow, Russia) following the manufacturer’s instructions, and their concentrations were measured using NanoDrop (Thermo Fisher Scientific, Waltham, MA, USA). Sanger sequencing was carried out using BigDye Terminator v3.1 reagents and SeqStudio, Aplied Biosystems (Thermo Fisher Scientific, Waltham, MA, USA) at the Research Platform for Agricultural Biotechnology, S.Seifullin Kazakh AgroTechnical Research University, Astana (Kazakhstan). SNPs were identified using manual comparison of the visualized sequences using the Chromas computer software program, version 2.0. The identified SNP were verified by sequencing of the same PCR products in both directions.

### 2.6. ASQ Plant Genotyping

The ASQ method was used for plant genotyping based on the previously published protocol [37] with the following modifications. The molecular probe with a short 4-bp tag was used, and two allele-specific forward primers together with one reverse primer were designed for two SNP, *LcSOC1-b2*-SNP. The composition of the PCR cocktail for ASQ genotyping and sequences of the allele specific primers and universal molecular probes are presented in Supplementary Materials Table S5.

The primers and molecular probes were obtained from DNA Synthesis (Moscow, Russia). Each reaction had a 10-μL cocktail in total and was loaded in a 96-well microplate. Allele discrimination was determined using a QuantStudio-7 Real-Time PCR system instrument (Thermo Fisher Scientific, Waltham, MA, USA) with automatically recorded fluorescence. Amplification of FAM and VIC was checked and controlled, whereas SNP calling and genotyping results were determined in a post-run step with analysis of Real-Time dRn setting with minimal error rate determined using the inbuilt software of the qPCR instrument, version 1.7.2. Genotyping experiments were carried out with three individual plants (biological replicates) and results were validated with two repeated runs (technical replicates) for each lentil genotype. The accuracy was confirmed using ‘No template control (NTC)’ with sterile water instead of template DNA.

### 2.7. Statistical Treatment

Means, standard errors, and significance levels were calculated using unpaired *t*-test, ANOVA, *F*-test with two-samples for variances, non-parametric Kruskal–Wallis and Mann–Whitney *U*-tests, and Pearson’s correlation functions based on software packages of Excel 365, Microsoft. At least three biological replicates (individual plants) and two technical repeats (instrumental runs) were used for each genotype and experiment.

## 3. Results

### 3.1. Scanning Electron Microscopy (SEM) of Internode Epidermal Cells

**Figure 2:**
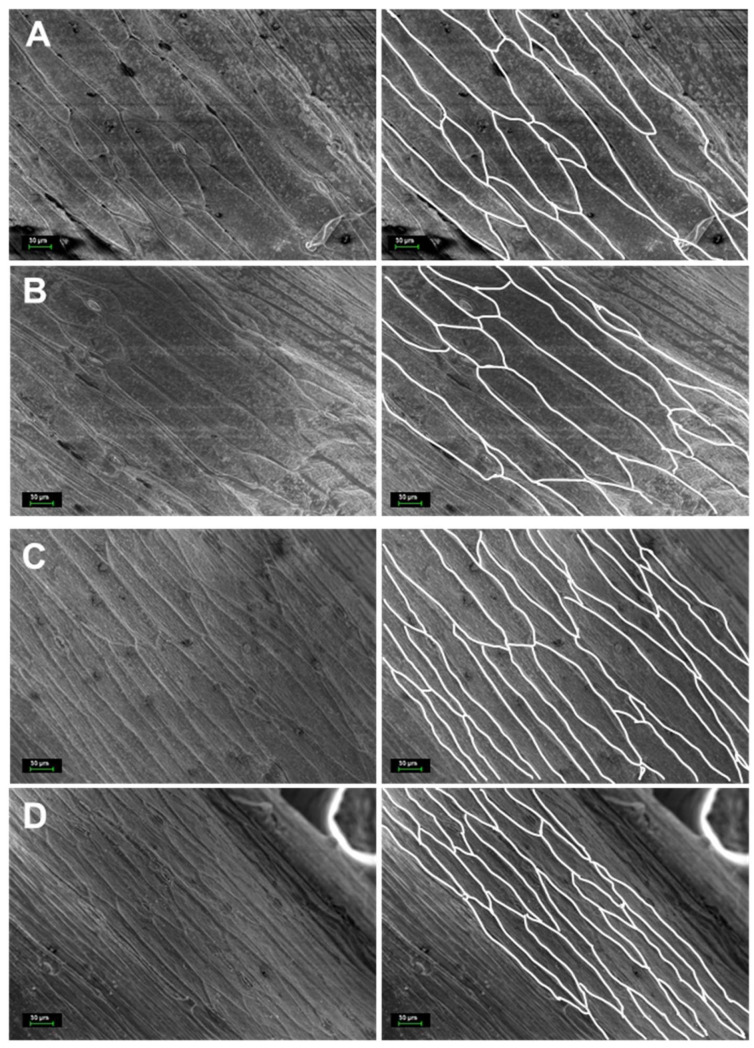
Scanning microscope images of the epidermal cells on the gold coated internodes below the lateral branch with first pods in two separate plants in each of two lentil accessions: (**A**,**B**) Flip92-36L with long internodes and HFP (13.0 cm); and (**C**,**D**) ILL-1552 with short internodes and HFP (6 cm). Images on the right represent copies of the originals on the left but with outlines of cell-wall boundaries to simplify measurements of cell length and width. The scale bar (30 µm) is indicated at the left bottom of each image.

SEM analysis was carried out on epidermal cells of stem internodes of two lentil accessions, Flip92-36L and ILL-1552, with long and short HFP, respectively (Figure 2). Measurement of SEM images revealed that the length of cells in the epidermis of internodes in both lentil accessions was almost identical, whereas width of the same epidermal cells was significantly larger (1.7-fold) in Flip92-36L plants compared to those in ILL-1552 (Table 1). In other words, plants of the lentil accession with long HFP have significantly wider epidermal cells in internodes compared to the narrower epidermal cells in plants of the lentil accession with short HFP. Cell length in the lentil accessions remained very similar (Table 1 and Figure 2).

### 3.2. Molecular Phylogenetic Analysis of LcSOC1 Genes in Lentil and Other Legumes

**Figure 3:**
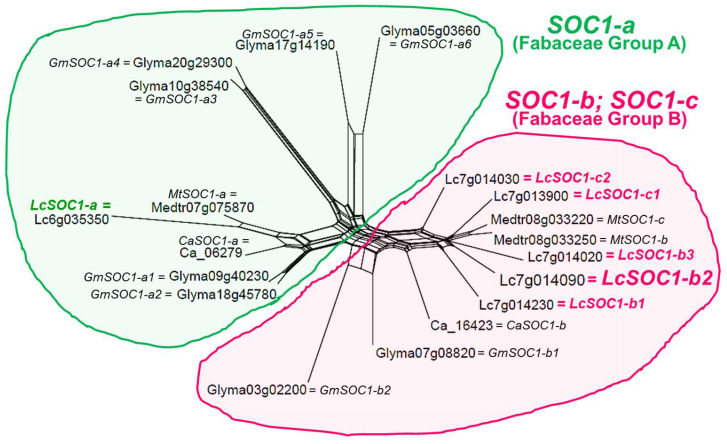
Molecular phylogenetic tree for *SOC1* genes in lentil and closely related legumes based on amino acid sequences of encoded polypeptides. Two clades, Fabaceae Group A and B, for *SOC1-a* and *SOC1-b/SOC1-c*, are designated by green and pink colour, respectively. Names of the genes and their identification in the genome correspond to the legume species: lentil, Lc, *Lens culinaris* Medik.; barrelclover, Mt, *Medicago truncatula* Gaertn.; soybean, Gm, *Glycine max* (L.) Merr.; and chickpea, Ca, *Cicer arietinum* L. The nomenclature of the *SOC1* genes was based on those in *M. trancatula* [31,32,33]. *LcSOC1* genes are designated by colour and the gene *LcSOC1-b2* selected for this study is indicated by the larger font. The molecular dendrogram was constructed using the SplitsTree4 program [55]. The full sequence of the legume *SOC1* genes and encoded proteins are presented in Supplementary Materials Table S2.

Based on publicly available lentil genome data in *Lens culinaris*, cv. Redberry (version gnm2. ann1) [53], six *LcSOC1* genes were identified and their encoded polypeptides showed strong similarity in phylogenetic analysis with other closely related legumes, soybean, chickpea and *M. truncatula* (Figure 3).

The first clade of the dendrogram, Fabaceae Group A, contains only the *SOC1-a* group of genes, with one gene each for lentil, *M. truncatula* and chickpea and two genes from soybean, *GmSOC1-a1* and *GmSOC1-a2*, clustered together with similar protein amino acid sequences. This clade has only four additional genes in soybean, from *GmSOC1-a3* to *GmSOC1-a6*, indicating multiple duplication in the soybean genome.

In contrast, the second clade of the phylogenetic tree, Fabaceae Group B, comprises two sub-groups of the relatively close *SOC1-b* and *SOC1-c* genes. Starting from the earliest described genes in *M. truncatula, MtSOC1-b*, it was accompanied by three genes from lentil, designated as *LcSOC1-b1*, *LcSOC1-b2* and *LcSOC1-b3*, respectively, followed by the single chickpea gene, *CaSOC1-b*, and two genes from soybean, *GmSOC1-b1* and *GmSOC1-b2*. On the other hand, a *MtSOC1-c* gene from *M. truncatula* showed similarity with only two lentil genes, *LcSOC1-c1* and *LcSOC1-c2* and encoding proteins (Figure 3).

### 3.3. Differential Expression of Six LcSOC1 Genes During Lentil Plants Development

**Figure 4:**
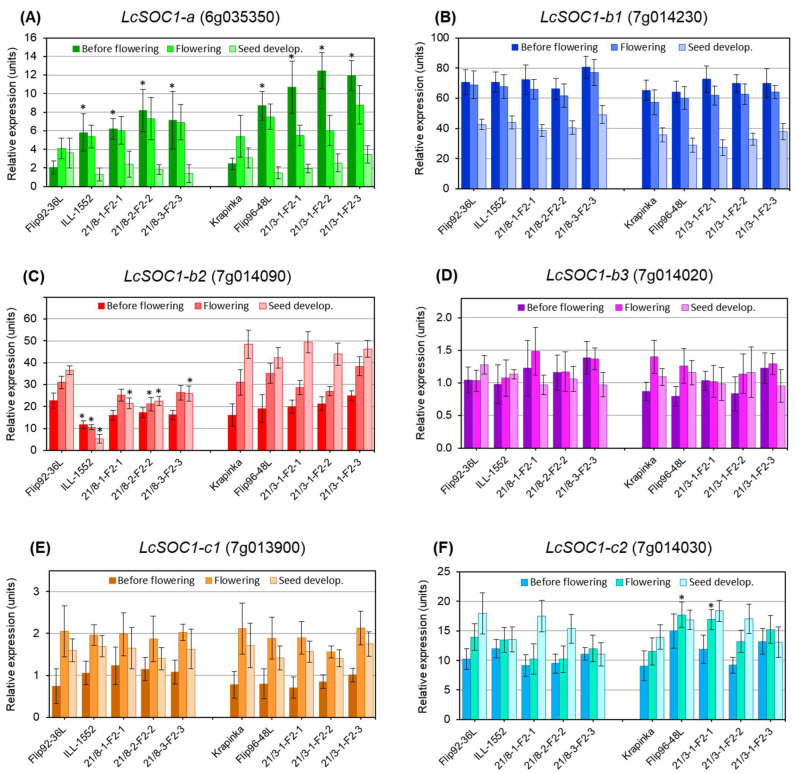
RT-qPCR expression analysis of six *LcSOC1* genes during plant development of parents and hybrid breeding lines from two hybrid populations: (**A**) *LcSOC1-a*; (**B**) *LcSOC1-b1*; (**C**) *LcSOC1-b2*; (**D**) *LcSOC1-b3*; (**E**) *LcSOC-c1*; and (**F**) *LcSOC1-c2*. On the left side of each panel, parents [♀Flip92-36L and ♂ILL-1552] and three breeding lines 21/8 represent hybrid 1, while parents [♀Krapinka and ♂Flip96-48L] and other three breeding lines 21/3, on the right side, are belonging to hybrid 2. Three developmental stages were used for sampling, indicated on the top of each panel and representing vegetative stage (before flowering), flowering and post-flowering (pods and seeds developments). Expression data were normalised using two reference genes, *LcELF4A-III* (Eukaryotic initiation factor 4A-III) and *LcActin-7*, and these data are present as the average ± SE of three biological replicates (individual plants) and two technical repeats for each genotype and developmental stage. Significant differences (* *p* < 0.05) between genotypes compared to maternal parent for each developmental stage in each of corresponding hybrid were calculated using two-way ANOVA with post-hoc Tukey test.

RT-qPCR expression analysis of the six identified *LcSOC1* genes was carried out in hybrid 1 [♀Flip92-36L × ♂ILL-1552] and hybrid 2 [♀Krapinka × ♂Flip96-48L], including their parents and three selected hybrid breeding lines for each cross, 21/8 and 21/3, respectively (Figure 4).

Most of the genes examined showed constitutive expression during vegetative growth, flowering and seed development stages. However, the mRNA level was diverse between the genes in this experiment. For example, very high expression was found in the *LcSOC1-b1* gene with a 60–80-fold higher level than the reference genes during the vegetative and flowering period followed by a reduction by almost half in the level of expression in all the lentil hybrids and genotypes (Figure 4B). In contrast, expression of *LcSOC1-b3* fluctuated around levels of reference genes in all stages of plant development and in all genotypes (Figure 4D). Two other genes, *LcSOC1-c1* and *LcSOC-c2*, showed rising expression profiles from level 1 to 2 to level 10 to 17, respectively, in almost all studied lentil genotypes after transition to flowering (Figure 4E,F).

Differential expression was found in the *LcSOC1-a* gene which, in particular, showed significant difference in the vegetative stage (Figure 4A). Both paternal genotypes, ILL-1552 and Flip96-48L, from hybrids 1 and 2, showed 3–4-fold higher mRNA production from the *LcSOC1-a* gene compared to the maternal parents, Flip92-36L and Krapinka, respectively. However, at flowering and seed setting stages, no further significant differences in gene expression were recorded, the levels returning to the common expression level in all parents and hybrid lines (Figure 4A). Therefore, differential expression of the *LcSOC1-a* gene was observed in the vegetative stage and only in early flowering lentil genotypes. This could indicate the involvement of this gene in the control of FT and the transition of plants from vegetative stage to flowering.

Only one gene, *LcSOC1-b2*, showed differential expression in the paternal genotype ILL-1552 in hybrid 1, with lowest HFP, and mRNA level of the genes was significantly, 2–4-fold, lower compared to the maternal parent Flip96-48L in all three developmental stages used in the experiment (Figure 4C). The three hybrid breeding lines 21/8 showed intermediate expression level with variability among the genotypes. In contrast, no significant differences were recorded for the expression of *LcSOC1-b2* between parents and their breeding lines in hybrid 2 (Krapinka × Flip96-48L), showing constant growing levels of the produced *LcSOC1-b2* mRNA from vegetative to reproductive stages in lentil genotypes (Figure 4C).

### 3.4. SNP Identification in LcSOC1-b2 Gene in Lentil Accessions

**Figure 5:**
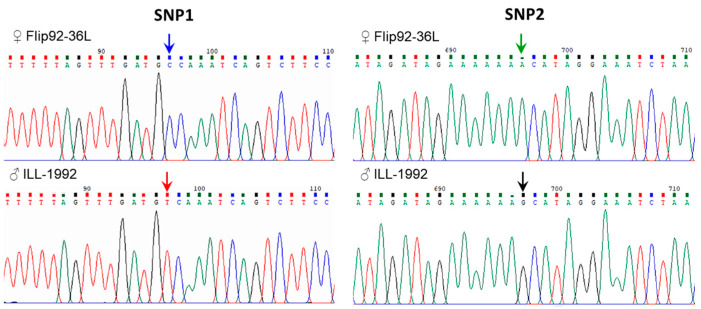
Fragments of Sanger sequencing of the first intron of the gene *LcSOC1-b2* (7g014090) in two lentil accessions used as parents for hybridization, maternal (Flip92-36L) and paternal (ILL-1552). Positions of two identified SNP (SNP1 and SNP2) are shown by arrows with different colours.

Strong conservation was found during sequencing of the *LcSOC1-b2* genes from eight lentil accessions with diverse origins. However, two SNP were identified in the first (and largest) intron, but none in other parts of the gene (Figure 5 and Supplementary Materials Table S6).

Relatively rare alleles ‘T’ in SNP 1 (Y = C/T) and allele ‘G’ SNP2 (R = A/G) of the *LcSOC1-b2* gene, represent one haplotype-A, and they were identified in two lentil accessions, ILL-1552 (ICARDA, Syria) and Vekhovskaya-1 (Russia). Additionally, the fully sequenced and publicly available lentil cv. Redberry (Canada) also showed the same *LcSOC1-b2* haplotype-A indicating that it may not be such a rare case. However, the other six accessions had haplotype-B with ‘C’ and ‘A’ alleles in SNP1 and SNP2, respectively (Figure 5 and Supplementary Materials Table S7). These six lentil accessions with *LcSOC1-b2* haplotype-B include three parents (Flip92-36L, Flip96-48L and Krapinka) and three other accessions (Lebanese Local, Syrian Local and Niva-95).

It is also important to emphasize that the two parents of hybrid 1, ♀Flip92-36L and ♂ILL-1552, were polymorphic for *LcSOC1-b2* SNP alleles making it possible to use this hybrid population for genotyping and further analyses. In contrast, parents of the second hybrid, ♀Krapinka and ♂Flip96-48L, were monomorphic for *LcSOC1-b2* and, therefore, not suitable for the genotyping study.

### 3.5. SNP Genotyping of Lentil Plants with ASQ Molecular Markers for the LcSOC1-b2 Gene and Marker-Trait Associations (MTA) with HFP

**Figure 6:**
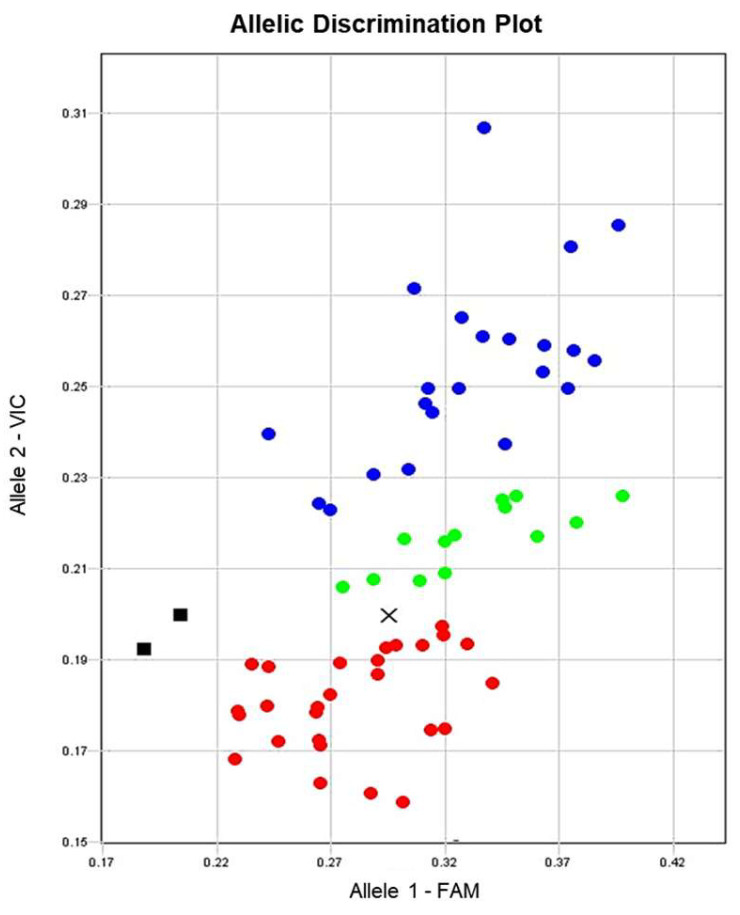
Example of plant genotyping for *LcSOC1-b2* alleles SNP1 in the lentil germplasm collection. The allele discrimination plot shows results of RFU (Relative fluorescence units) for FAM and VIC signals, assigned in X- and Y-axes of the figure using ASQ molecular marker *LcSOC1-b2-*SNP1. Red and blue dots represent homozygote genotypes *aa* and *bb* for alleles 1 (FAM) and allele 2 (VIC), respectively. Green dots indicate fluorescence signals of both FAM and VIC estimated as heterozygotes *ab* or an admixture of several genotypes. The allele discrimination was based on dRn automatic SNP calling. The black square shows the no template control (NTC) using water instead of template DNA, whereas a cross indicated a genotype with undetermined status.

Two ASQ molecular markers were developed (Supplementary Materials Table S5) and used successfully in targeting both SNP1 and SNP2 in *LcSOC1-b2* with reference genotypes Flip92-36L and ILL-1552, parents of hybrid 1. Plants of three selected breeding lines 21/8 from F_3_ progeny in the hybrid 1 were all heterozygotes and inherited *LcSOC1-b2* alleles from both parents. One ASQ marker, *LcSOC1-b2-*SNP1, with the best performance was selected and used for plant genotyping from the lentil germplasm collection. The genotyping results showed clear distribution of *LcSOC1-b2* alleles SNP1 (Figure 6).

All sequenced reference genotypes were confirmed for their status of the SNP1-allele during genotyping with *LcSOC1-b2-*SNP1 ASQ marker. However, a relatively large number of the genotypes showed fluorescence of both FAM and VIC, indicated by green dots in Figure 6. This can be related to the possible admixture or heterozygotes ‘*ab*’ SNP1 of the *LcSOC1-b* gene. Their genotypes have to be confirmed in further developed progenies.

The lentil germplasm collection with 66 accessions as listed in Supplementary Materials Table S1 was checked for *LcSOC1-b2*-SNP1 genotyping. However, six accessions failed to produce acceptable and reproducible amplification during ASQ genotyping, and they were eliminated from further analysis. For the remaining 60 accessions, marker-trait association (MTA) was studied between plant genotyping with the ASQ marker *LcSOC1-b2*-SNP1 and their phenotyping scores for HFP. The genotyping was carried out in 96-well microplate as described above, whereas HFP and FT were measured in plants grown in field trials.

Results for MTA indicated a strong association between *LcSOC1-b2*-SNP1 marker and HFP (Table 2). Non-parametric statistical analysis using the Kruskal–Wallis test resulted in highly significant (*p =* 0.00223, *p* < 0.01) differences between the three groups of genotypes with their HFP measurements. However, only homozygotes *aa* and *bb* were significantly different from the total number of 60 lentil accessions using ASQ marker *LcSOC1-b2*-SNP1, whereas no such difference was found in the case of heterozygotes *ab* (Table 2). Results of statistical treatments using non-parametric Mann–Whitney *U*-test with unequal variances showed differences between homozygotes of *LcSOC1-b2*-SNP1 alleles (Table 2). Therefore, two groups of lentil genotypes with *aa* and *bb* homozygotes for the SNP1 marker showed highly significant association with HFP phenotyping.

Marker-trait association was also checked between ASQ marker *LcSOC1-b2*-SNP1 genotyping and FT trait measurements. However, no significant associations were found in any group of genotypes, homo- or heterozygotes for *LcSOC1-b2*-SNP1 alleles with FT. This was confirmed by a Kruskal–Wallis test (*p* = 0.3092) showing no significant differences between three groups of genotypes with their FT measurements in days to flowering. Similar results were provided by Mann–Whitney *U*-test (two-sample with unequal variances), where no association was found between FT trait and *LcSOC1-b2*-SNP1 alleles from the three groups of 60 studied lentil genotypes (Table 2).

These results indicate that *LcSOC1-b2* is not directly involved in the control of transition of lentil plants from vegetative to reproductive stage and time to flowering.

## 4. Discussion

The nature of the height to first pod trait in lentils is not as simple as first expected. It is logical to conclude that elongation of stem cells will result in longer internodes [8,62]. GA and GA-related genes are well-known enhancers of cell elongation [6,63], capable of increasing the rate of cell elongation in both intact plants and excised segments [6]. GA was reported to induce cell elongation in lettuce hypocotyls (*Lactuca saliva* L.) [64], and in early stages, light-grown hypocotyls of *Arabidopsis thaliana* [65]. In *Brassica* mutants, endogenous GA content was associated with cell length, and the application of exogenous GA to normal plants increased cell length [66]. In potato (*Solanum tuberosum* L.), the cells in the sub-apical region of lateral shoots showed clear elongation in GA-treated segments [67]. In transgenic tobacco plants overexpressing *Arabidopsis* bZIP transcriptional activator (Repression of shoot growth), stem internode growth was severely inhibited, specifically for cell elongation, due to the transgene controlling the endogenous GA [7].

While studies mentioned above and many other have shown the importance of GA on cell elongation, in the present study, cell length did not account for increased height to first pod. Epidermal cells in stem internodes of two lentil accessions, Flip92-36L and ILL-1552, with high and low HFP (13 and 6 cm), respectively, were found to have practically identical cell length under SEM (Table 1 and Figure 2). GA-related genes were not considered in our study because cell length was not involved in determining HFP in the lentil germplasm. Additionally, no published evidence was found that GA-related genes or GA-treatment can be associated with equal or unchanged cell length in plant stem compared to controls or untreated plants.

Similarly, there are several reports that other genes can control cell elongation and these genes encode various hormones like AUX [9,10] or BS [13]. However, longer or shorter stem internodes with no differences in cell length were found only in two papers, where the number of cells could determine the internode length [11,19]. The first case involves the *BR2* gene encoding AUX efflux P-glycoprotein-1 in the maize *br2* mutant, which had shorter internodes, but the same cell size compared to WT [11]. The second example was regarding the grapevine ZJZS mutant, which had very short stem internodes compared to WT but with very similar cell length, and the authors reported on genes involved in JA biosynthesis and metabolism [19]. Nevertheless, the mechanisms of cell proliferation via more cell division rather than cell elongation in these examples are very different and there is no evidence for any interaction or network links between AUX efflux *BR2* genes [11] and *TCP* TF genes regulating JA [19,20,21]. Moreover, in the current study, *SOC1* MADS-box TF genes appear to represent a third mechanism unlinked to the first two. The reason for multiple and independent mechanisms controlling cell proliferation, and division with unchanged cell length, in plant stems remains unclear. Therefore, more analysis is required in further experiments.

The process of cell growth and division in stems of different plant species could be related to one of three (or more) genetic mechanisms with complicated interacting networks, i.e., AUX, JA or SOC1 polypeptides. Therefore, for the purpose of our study, it was important to determine which regulatory genes might be involved in the control of length, width and number of stem cells associated with longer or shorter internodes in lentil plants.

Based on published reports, *SOC1* genes were shown to control FT and transition of plants to flowering, but some of the *SOC1* genes are actively involved in HFP in *M. truncatula* [31,32]. However, while members of the *SOC1* gene family are diverse and sometimes overlapping in their functions in legumes [25,26], they are nonetheless can play very different role in other species [27,28,30]. Our expression analysis of six identified *LcSOC1* genes indicated very large differences in the magnitude and pattern of expression from vegetative to reproductive stages (flowering and seed setting) (Figure 4).

*LcSOC1-a* was differentially expressed in all lentil genotypes, parents, and breeding lines in vegetative tissue and until flowering, but dropped significantly after, similar to earlier published results on *MtSOC1-a* in *M. truncatula* [31,33].

Two of the three *LcSOC1-b* genes, *LcSOC1-b1* and *-b2*, also have some functional similarity with *MtSOC1-b*, involved in plant flowering, but this gene was also expressed in stems and likely participates in internode elongation in *M. truncatula* [33,34]. Different and separate functions of *LcSOC1-b1* and *-b2* genes were found in lentil genotypes. *LcSOC1-b1* showed high and consistent initial expression levels but reduced by two-fold during seed setting in all lentil accessions. This gene is likely related to transition to flowering in lentil plants, as demonstrated earlier [35]. The expression of this gene was very high in lentil plants in response to light quality (high ratio of red/far red light), which is important for flowering of lentil plants.

In contrast, the second gene, identified in our study, *LcSOC1 b2,* showed clear and substantially differing expression profiles in lentil parents, Flip92-36L and ILL-1552, with contrasting (long and short) HFP and their breeding lines. Additionally, this gene was not related to FT and the transition of lentil plants to flowering in our study. Other *LcSOC1* genes showed no changes in expression level, remaining consistently at a low, moderate or high level (Figure 4).

Two SNP-based haplotypes in the *LcSOC1-b2* gene were identified here, and genotyping using the *LcSOC1-b2*-SNP1 marker showed a distribution among the lentil accessions (Figure 5 and Figure 6). This finding is novel; neither *LcSOC1-b2* gene had been identified earlier, nor their haplotypes and genetic polymorphism described here. The haplotype-A includes the reference genotype of lentil, cv. Redberry (Canada), parent ILL-1552 (ICARDA), and 22 accessions in total including mostly accessions from ICARDA and VRIPGR, with average HFP for 11.73 cm (Table 2). In contrast, haplotype-B was found in 27 lentil accessions with an average HFP of 15.58 cm, including most of the cultivars from Kazakhstan and Russia, as well as accessions from VRIPGR, but only a few from ICARDA (Table 2).

This study suggests that *LcSOC1-b2* could be one of the major regulators involved in the HFP trait in lentil. Other potential co-regulators remain unclear. They might be genes involved in JA metabolism, AUX or BS hormones or combinations of any of these. These coordinated responses may be realised into cell division and increasing cell numbers, but not elongation of cells in stem internodes of lentil plants.

## Figures and Tables

**Table 1:** Length and width of epidermal cells in stem internodes of two lentil accessions with high and low HFP (*n* = 30, for each genotype), using scanning microscopy analysis. Significance was calculated using *F*-test, two-samples for variances.

Accessions	Cell Length (µm)		Cell Width (µm)	
	Average	SE ^1^	Average	SE
Flip92-36L	270.8	24.7	47.1	3.7
ILL-1552	267.4	18.1	27.2	1.7
*Significance*	n.s. ^2^		*** *p* < 0.001	

^1^ SE, Standard error; ^2^ n.s., not significant.

**Table 2:** Marker-trait association between genotyping of the germplasm collection using ASQ marker *LcSOC1-b2*-SNP1 and phenotyping of plants for HFP and FT. Significance of the differences between genotypes in each group and the total were estimated using non-parametric Mann–Whitney *U*-test (MWU-test).

Genotype	Fluoro- Phore	*n*	HFP (cm)		HFP MWU-Test with Total	FT (Days)		FT MWU-Test with Total
			Average	SE ^1^		Average	SE	
***aa***	**FAM**	22	11.73	0.76	*p* = 0.0222, ** *p* < 0.1	39.68	0.33	*p* = 0.3087, n.s.
***ab***	**FAM/VIC**	11	14.18	0.96	*p* = 0.8267, n.s. ^2^	40.18	0.49	*p* = 0.7910, n.s.
***bb***	**VIC**	27	15.58	0.65	*p* = 0.0546, ** *p* < 0.1	40.27	0.29	*p* = 0.4499, n.s.
*Total*		60	13.88	0.48	-	40.03	0.20	-

^1^ SE, standard error; ^2^ n.s., not significant. Colours of the presented genotypes correspond to fluorophores: ‘*aa*’ and FAM are in red, whereas ‘*bb*’ and VIC are in blue. Heterozygote genotypes ‘*ab*’ are shown in green and correspond to both fluorophores, FAM and VIC. The colour identifications of the genotypes and fluorophores are the same as present in Figure 6 above.

## Data Availability

The original contributions presented in this study are included in the research paper and in Supplementary Materials. Further inquiries can be directed to the corresponding authors.

## Supplementary Materials

The following supporting information can be downloaded at: https://www.mdpi.com/article/10.3390/plants14081157/s1, Table S1: a list of 66 studied lentil germplasm; Table S2: sequences of the identified *SOC1* genes and encoded proteins in the studied legume species; Table S3: sequence of gene-specific primers and primers for reference genes used for qPCR analysis in the study; Table S4: the designed and used primers for Sanger sequences indicated in genome fragment with entire *LaSOC1-b2* (Lcu.2RBY.7g014090) gene in lentil; Table S5: ASQ genotyping: PCR cocktail composition, sequences of the used allele specific primers and universal molecular probes; Table S6: genome sequence of *LcSOC1-b2* gene with indicated two SNP in studied lentil accessions; Table S7: haplotypes of *LcSOC1-b2* gene based on SNP1 and SNP2 in eight studied lentil accessions.
