# Supplementary material for: Longer Internode with Same Cell Length: LcSOC1-b2 Gene Involved in Height to First Pod but Not Flowering in Lentil (Lens culinaris Medik.)
Source: Plants (Basel). 2025 Apr 8;14(8):1157. doi: 10.3390/plants14081157 (PMC12030134; doi:10.3390/plants14081157)
Supplement: Supplementary file 1 [file plants-14-01157-s001.zip › plants-3528735-supplementary.pdf]

Supplementary Materials

Kuzbakova M., Khassanova G., Jatayev S., Daniyeva N., Sweetman C., Jenkins C.L.D., Soole K.L., Shavrukov Y.

Longer Internode with Same Cell Length: *LcSOC1-b2* Gene Involved in Height to First Pod but Not Flowering in Lentil (*Lens culinaris* Medik.)

Table S1. A list of the 66 studied lentil germplasm.

Alphabetical order					Arranged by genotyping for *LcSOC1-b2*			
No	Name	Origin	HFP (cm)	Geno-type	Name	Origin	HFP (cm)	Geno-type
1	Chiflik	Bulgaria	16	*ab*	K-2846, Roze	Canada	18	*aa*
2	D31	Canada	16	*bb*	K-2583	Azerbaijan	17	*aa*
3	Flip86-51L	ICARDA	9	*aa*	Shyraily	Kazakhstan	17	*aa*
4	Flip89-63L	ICARDA	9	*aa*	K-34983	Hungary	16	*aa*
5	Flip90-25L	ICARDA	9	*aa*	K-468	Armenia	15	*aa*
6	Flip90-41L	ICARDA	9	*bb*	K-5883, 81S15	Georgia	15	*aa*
7	Flip92-36L	ICARDA	13	*bb*	K-2720	Columbia	14	*aa*
8	Flip95-30L	ICARDA	12	*ab*	K-2708, E-157	Ecuador	13	*aa*
9	Flip96-15L	ICARDA	9	*ab*	ILL-4611	Nepal	12	*aa*
10	Flip97-6L	ICARDA	8	*aa*	PI-435960	Iran	12	*aa*
11	ILL-474	Syria	10	*bb*	PI-509335	Türkiye	12	*aa*
12	ILL-1464	Iran	10	*aa*	PI-543920	USA	11	*aa*
13	ILL-1552	Iran	6	*aa*	PI-468898	Brazil	10	*aa*
14	ILL-4611	Nepal	12	*aa*	ILL-1464	Iran	10	*aa*
15	ILL-5725	Syria	8	*aa*	Flip86-51L	ICARDA	9	*aa*
16	K-188	Georgia	14	*ab*	Flip89-63L	ICARDA	9	*aa*
17	K-192	Azerbaijan	18	*bb*	Flip90-25L	ICARDA	9	*aa*
18	K-408	Palestine	18	*bb*	Sel97-39L	ICARDA	9	*aa*
19	K-468	Armenia	15	*aa*	Flip97-6L	ICARDA	8	*aa*
20	K-474	Armenia	15	*ab*	ILL-5725	Syria	8	*aa*
21	K-482	Armenia	14	*bb*	PI-557499	USA	8	*aa*
22	K-660	Azerbaijan	13	*bb*	ILL-1552	Iran	6	*aa*
23	K-662	Azerbaijan	20	*bb*	K-2834, PR-86-385	Canada	19	*ab*
24	K-664	Azerbaijan	15	*bb*	K-883	Palestine	18	*ab*
25	K-883	Palestine	18	*ab*	Chiflik	Bulgaria	16	*ab*
26	K-894	Canada	18	*bb*	K-474	Armenia	15	*ab*
27	K-903, Mestniy	Russia	18	*bb*	K-2715	Ecuador	15	*ab*
28	K-907	Armenia	20	*bb*	K-188	Georgia	14	*ab*
29	K-1083	Italy	13	*bb*	K-2796	Ecuador	14	*ab*
30	K-1084	Italy	14	*bb*	Vekhovskaya-1	Kazakhstan	14	*ab*
31	K-2127, Rozovaya	Russia	13	*bb*	Flip95-30L	ICARDA	12	*ab*

32	K-2583	Azerbaijan	17	*aa*	PI-509334	Türkiye	10	*ab*
33	K-2589, Mestniy	Armenia	17	*bb*	Flip96-15L	ICARDA	9	*ab*
34	K-2707	Mexico	22	*bb*	K-2707	Mexico	22	*bb*
35	K-2708, E-157	Ecuador	13	*aa*	K-662	Azerbaijan	20	*bb*
36	K-2713, E-112	Ecuador	18	*bb*	K-907	Armenia	20	*bb*
37	K-2715	Ecuador	15	*ab*	K-2716, Procor	Brazil	19	*bb*
38	K-2716, Procor	Brazil	19	*bb*	K-192	Azerbaijan	18	*bb*
39	K-2717	Mexico	16	*bb*	K-408	Palestine	18	*bb*
40	K-2720	Columbia	14	*aa*	K-894	Canada	18	*bb*
41	K-2721	Columbia	15	*bb*	K-903, Mestniy	Russia	18	*bb*
42	K-2796	Ecuador	14	*ab*	K-2713, E-112	Ecuador	18	*bb*
43	K-2834, PR-86-385	Canada	19	*ab*	K-2589, Mestniy	Armenia	17	*bb*
44	K-2846, Roze	Canada	18	*aa*	D31	Canada	16	*bb*
45	K-5883, 81S15	Georgia	15	*aa*	K-2717	Mexico	16	*bb*
46	K-34983	Hungary	16	*aa*	Niva-95	Russia	16	*bb*
47	Niva-95	Russia	16	*bb*	K-664	Azerbaijan	15	*bb*
48	Petrovskaya	Russia	15	*bb*	K-2721	Columbia	15	*bb*
49	PI-435960	Iran	12	*aa*	Petrovskaya	Russia	15	*bb*
50	PI-468898	Brazil	10	*aa*	Vekhovskaya	Kazakhstan	15	*bb*
51	PI-509334	Türkiye	10	*ab*	K-482	Armenia	14	*bb*
52	PI-509335	Türkiye	12	*aa*	K-1084	Italy	14	*bb*
53	PI-543920	USA	11	*aa*	Flip92-36L	ICARDA	13	*bb*
54	PI-557499	USA	8	*aa*	K-660	Azerbaijan	13	*bb*
55	Precoz	Argentina	10	*bb*	K-1083	Italy	13	*bb*
56	Richelea	Canada	13	*bb*	K-2127, Rozovaya	Russia	13	*bb*
57	Sel97-39L	ICARDA	9	*aa*	Richelea	Canada	13	*bb*
58	Shyraily	Kazakhstan	17	*aa*	ILL-474	Syria	10	*bb*
59	Vekhovskaya	Kazakhstan	15	*bb*	Precoz	Argentina	10	*bb*
60	Vekhovskaya-1	Kazakhstan	14	*ab*	Flip90-41L	ICARDA	9	*bb*

Not genotyped

61	Flip87-56L	ICARDA	8
62	K-924	Syria	13
63	K-2601	Mexico	15
64	K-2843, 89ZPR-8	Canada	14
65	K-2845, 89-12	Canada	16
66	PI-509330	Türkiye	9

<u>Table S2</u>. Sequences of the identified *SOC1* genes and encoded proteins in the studied legume species.

<u>CDS</u>

>LcSOC1-a. Lencu.CDC_Redberry.gnm2.ann1.Lcu.2RBY.6g035350.1 (468bp)

AGCATGCAGGACACAATTGAACGGTACCGCAGAAATACAAGGAGTGCTCAACCAGTGCAAAGATCAG
ATGAACAAAATATGCAGAGTTTGAAGCTAGAAACAGCAAGTTTGATGAAGAAGATTGAGATTCTCGA
AGCTTCGAAACGGAGACTCATGGGAGAAGGTTTAGGTTCATGCTCCTTGGATGAACTGCAACAGATA
GAACAACAGTTGGAAAAAAGTGTAAGCACTGTTCGAGCAAGAAAGAATCAGGTTTACAAGAATCAAA
TTGAGCAACTTAAAGAAAAGGAAAAAGCCTTACTTGCTGAAAATTCCAGACTCTCTAAGCAGCCGCA
GCCGCAGCCAGTGCCACCGCCAGTGCCACCGACAAATGATCGTCCGAGAGATAATCAAGCGTATGCA
GAAAGCAGCAGTCCAAGCTCGGGCGTGGTGACTGAATTGTTCATTGGACTACACAGGTCTAATTGA

>LcSOC1-b1. Lencu.CDC_Redberry.gnm2.ann1.Lcu.2RBY.7g014230.1 (774 bp)

ATGTCAAACTATAAGACTCAACCCTTTATTCCTATCTCTTCTCTCATTCATTTTCTTCTCTATTTTG
GTGCAAATCTGTTTCCTCTTGAAGTTGCTTTTCTTGCTGGAAAGATGGTGAGAGGGAAGACAGAAAT
GAAGCGTATAGAGAACGCAACAAGTAGGCAAGTGACATTTTCAAAGAGAAGAAACGGTTTGATGAAG
AAAGCTTTTGAGTTATCAATTTTGTGTGATGCTGAAGTTGCTCTTATTGTTTTTTCACCAAGAGGGA
GAGTTTATGAATTTGCAAGTTCAAGCATTTTAGAAACAATTGATCGATACCGCAGTCATACAAAGAA
TCATAATACTCCAACAACATCTGAATCTGCTGAAAATACTCAGCGTTTGAAGGAAGAAGCAGAAAAC
ATGATGAAAAAAATTGATCTTCTTGAGACTTCTAAACGAAAACTCTTAGGAGAAGGTTTAGGGACTT
GTTCCATGGATGAACTACAAAGGGTAGAGCAACAGTTGGAGAGGAGTATAGCCAAAATTCGTCTTAA
AAAGACCGAAGTTTTCAGAGAACAAATTGAACTGCTCAAAGAAAAGGAAAAAACCCTAACTGCTGAA
AATACCAGGCTCTCTGAGAAGGAAGCAAAAAAGGATGATAGAGAAAATACAGGTGAGGTTGAAGGTT
ATGCAGATGAAAGTAGTCAAAATTCAGATGTGGAGACTGAATTATTCATTGGTCTTCCAGAAACAAG
AACAAGGCGAATTTCTCCCAAGTTGAGGACTAATTAA

>LcSOC1-b2. Lencu.CDC_Redberry.gnm2.ann1.Lcu.2RBY.7g014090.1 (684 bp)

ATGGTGAGAGGGAAGACACAGATGAAGCGTATAGAGAACGCAACAAGCAGACAAGTAACATTTTCAA
AGAGAAGAAATGGTTTGATGAAGAAAGCTTTTGAGTTATCAGTTTTGTGTGATGCTGAAGTTGCTCT
TATTGTTTTTTCATCAAGAGGGAGACTTTATGAATTTGCAAGCTCAGGCATTCTAGAAACAATTGAA
CGATACCGCAGTCATAGCCGGATTAATAATAATTCAACAACAACCGAATCTGCTGAAAATACTCAGC
ATTTGAAGGAAGAAGCAGAGAACATGATGAAAAAGATTGATCTTCTTGAGACTTCTAAACGAAAACT
CTTAGGAGAAGGTTTAGGAACTTGTTCCATTGATGAACTGCAAAGGATAGAGCAACAGTTGGAGAGG
AGTATAACCAAAATTCGAGTTAGAAAGGCTGAGGTTTTCAAAGAACAGATTGATCAGCTAAAAGAAA
AGGAAAAAACCCTAGTTGCTGAAAATACCAGGCTCTCTGAGAAGTATAATAACTATTCATCACAGCA
AGGAAAAAAGGATGATAGAGTAAATGTAGATGAGGTTGATGCTTATGCAGATCAAAGTAGTCCAAAT
TCAGATGTTGAAACTGAGTTATTCATTGGTCTTCCAGAAACAAGAACAAGGAAAATTTCTCCAATGT
TGAGGACTAACTAA

>LcSOC1-b3. Lencu.CDC_Redberry.gnm2.ann1.Lcu.2RBY.7g014020.1
(720 bp)

ATGGTGAGAGGGAAGACACAGATGAAGCGCATAGAGAACGCAACAAGTAGACAAGTAACATTTTCAA
AGAGAAGAAATGGTTTGATGAAGAAAGCTTTTGAGTTATCTATTTTGTGTGATGCTGAAGTTGCTCT
TATTGTTTTCTCACCAAGAGGAAGACTTTATGAATTTGCAAGCTCAAGCATTCTAGAAACAATTGAT
CGTTACCGCAATCGTAGTCAAATTAATAATACCTCAAGAACATCTGATTGTGATGAAAATACTCAGC
ATTTGAAGGAAGAAGCAGAAAACATGATGAAAAAGATTGATCTTCTTGAGACTTCAACGCGAAAACT
CTTAGGAGAAGGTTTAGGGACTTGTTCCATTGGAGAACTGCAAAAGATAGAGCAAGAGTTGGAGAGG
AGTATAACCAAAATTCGAGCAAAAAAGACTCAGGTTTACAGGGAGCAAATAGATCAGCTAAAAGAAA
AGGAAAAAGCCCTACTTGCCGAAAAGACTAGTCTCTCTGAGAAGTTATTATGGAACAATGAAAATCA
AATTCTTCTGCAGTGTGATAGTTATTCATCACAGCAAGCAAAAAAGGATGACAGGGAAAATATAGCT
CAGGCTGAACCTTCTGCAGATCAAAGTAGTCCAAGTTTAGACGTTGAAACTGAATTATTCATTGGTC
TCCCAGAAACAAGAACTAGAGGAATTTCTCCAAAGTTGAGGATTAAATAA

>LcSOC1-c1. Lencu.CDC_Redberry.gnm2.ann1.Lcu.2RBY.7g013900.1
(684 bp)

ATGGTGAGAGGGAAGACACAGATGAAGCGTATAGAGAACGCAACAAGTAGACAAGTAACATTTTCAA
AGAGAAGAAATGGTTTGATGAAGAAAGCTTTTGAGTTATCTATTTTGTGTGATGCTGAAGTTGCTCT
TATTGTTTACTCACCAACAGGAAGACTTTATGAATTTGCAAGTTCAAGCATTTCAGAAACAATTGAA
CGATACTGCAGTCATAGCAGGATTAATAATACTCCAACAACATCTGAATCTGCTGAAAATACTCAGC
ATTTGAGGGAAGAAGCAGAAAACATGATGAAAAAGATTGATCTTCTTGAGACTTCTAAACGGAAACT
CTTAGGAGAAGGCTTGGGGAGTTGTTCCATTGATGAACTACAAAAGATAGAGCAACAGTTGGAGAAT
AGTATAATCAAAGTTCGAGCGAAAAAGACTCAGGTTTTCAGGGAACAAATTCACCAGCTAAAAGAAA
AGGAAAAAACCCTAGTTGCTGAAAATATCTTGCTCTCGGAGAAGTATGATAACTATTCATCAAAGGC
AGCAAAAAACAATGAAAAAGAAAATATAGGTGAAGGTGAAGCTTATGCATATCAGAGTAGTCCAAGT
TCAGATGTGGAGACTGAATTGTTCATTGGTCTTCCAGAAACAAGAACAAGGAGAATTTCTCCAAACT
TGACGACTAATTAG

>LcSOC1-c2. Lencu.CDC_Redberry.gnm2.ann1.Lcu.2RBY.7g014030.1
(528 bp)

ATGGTGAGAGGAAAGACGCAGATGAAGCGTATAGAAGACGCAACAAGTAGGCAAGTAACATTCTCAA
AGAGAAGAAATGGTTTGATGAAGAAAGCTTTTGAGTTATCAATTTTGTGTGATGCTGAGGTTGCTCT
TATTGTTTTTTCACCAAGAGGGAGACTTTATGAATTTGCCAGCTCAAGCATTTTAGAAACAATTGAA
CGATACCGCAGTAATACAAGGATGAATAATACTCCAACAACATCTGAATCTGCTGAAAATACTCAGC
ATTTGAAGGAAGAAGTAGAAAACATGATGAAAAAGATTGAACTTCTTGAGACTTCAAAACGGAAAAT
TTTAGGAGAAGGTTTGGGGAGTTGTTCCATTGATGAACTACAAAAAATAGAGCAAAGGTTGGAGAAG
AATATAACCAAAATTCGAGAAAAAAAGACTCAGGTTTTTAAAGAACAAATTGACAAGCTAAAAGAAA
AGTCTCTCAAAGAAGAAAAAGTCGGGCGATTTGTCGAGAGCACAAAAAAGAAAAGATAA

<u>Proteins</u>

>Lc6g035350= LcSOC-a
SMQDTIERYRRNTRSAQPVQRSDEQNMQSLKLETASLMKKIEILEASKRRLMGEGLGSCSLDELQQI
EQQLEKSVSTVRARKNQVYKNQIEQLKEKEKALLAENSRLSKQPQPQPVPPPVPPTNDRPRDNQAYA
ESSSPSSGVVTELFIGLHRSN

>Lc7g014230=LcSOC-b1
MSNYKTQPFIPISSLIHFLLYFGANLFPLEVAFLAGKMVRGKTEMKRIENATSRQVTFSKRRNGLMK
KAFELSILCDAEVALIVFSPRGRVYEFASSSILETIDRYRSHTKNHNTPTTSESAENTQRLKEEAEN
MMKKIDLLETSKRKLLGEGLGTCSMDELQRVEQQLERSIAKIRLKKTEVFREQIELLKEKEKTLTAE
NTRLSEKEAKKDDRENTGEVEGYADESSQNSDVETELFIGLPETRTRRISPKLRTN

>Lc7g014090=LcSOC-b2
MVRGKTQMKRIENATSRQVTFSKRRNGLMKKAFELSVLCDAEVALIVFSSRGRLYEFASSGILETIE
RYRSHSRINNNSTTTESAENTQHLKEEAENMMKKIDLLETSKRKLLGEGLGTCSIDELQRIEQQLER
SITKIRVRKAEVFKEQIDQLKEKEKTLVAENTRLSEKYNNYSSQQGKKDDRVNVDEVDAYADQSSPN
SDVETELFIGLPETRTRKISPMLRTN

>Lc7g014020=LcSOC-b3
MVRGKTQMKRIENATSRQVTFSKRRNGLMKKAFELSILCDAEVALIVFSPRGRLYEFASSSILETID
RYRNRSQINNTSRTSDCDENTQHLKEEAENMMKKIDLLETSTRKLLGEGLGTCSIGELQKIEQELER
SITKIRAKKTQVYREQIDQLKEKEKALLAEKTSLSEKLLWNNENQILLQCDSYSSQQAKKDDRENIA
QAEPSADQSSPSLDVETELFIGLPETRTRGISPKLRIK

>Lc7g013900=LcSOC-c1
MVRGKTQMKRIENATSRQVTFSKRRNGLMKKAFELSILCDAEVALIVYSPTGRLYEFASSSISETIE
RYCSHSRINNTPTTSESAENTQHLREEAENMMKKIDLLETSKRKLLGEGLGSCSIDELQKIEQQLEN
SIIKVRAKKTQVFREQIHQLKEKEKTLVAENILLSEKYDNYSSKAAKNNEKENIGEGEAYAYQSSPS
SDVETELFIGLPETRTRRISPNLTTN

>Lc7g014030=LcSOC-c2
MVRGKTQMKRIEDATSRQVTFSKRRNGLMKKAFELSILCDAEVALIVFSPRGRLYEFASSSILETIE
RYRSNTRMNNTPTTSESAENTQHLKEEVENMMKKIELLETSKRKILGEGLGSCSIDELQKIEQRLEK
NITKIREKKTQVFKEQIDKLKEKSLKEEKVGRFVESTKKKR

>Medtr07g075870=MtSOC1-a
MVRGKTQMKRIENATSRQVTFSKRRNGLLKKAFELSVLCDAEVALIIFSPRGKLYEFSSSCSMQDTI
ERYRRNTRSAQPMQRSDEQNMQNLKHETASLMKKIELLEASKRKLMGEGLGSCSLDELQQIEQQLEK
SVSVVRARKNQAYKHQIDQLKEKEKNLVAENARLSKQPPQPQPQPTTKDHQREDQQPYAESSPSSDV
VTELFIGLHRSS

>Medtr08g033250=MtSOC1-b
MVRGKTQMKRIENATSRQVTFSKRRNGLLKKAFELSVLCDAEVALIVFSPRGRLYEFASSSILETIE
RYRSHTRINNTPTTSESVENTQQLKEEAENMMKKIDLLETSKRKLLGEGLGSCSIDELQKIEQQLEK
SINKIRVKKTKVFREQIDQLKEKEKALVAENVRLSEKYGNYSTQESTKDQRENIAEAEPYADQSSPS
SDVETELFIGLPETRSRRISPKV

>Medtr08g033220=MtSOC1-c
MVRGKTQMKRIENATSRQVTFSKRRNGLLKKAFELSVLCDAEVALIVFSPRGRLYEFTSSSILETIE
RYRSHTRINNTPTTSESVENTQQLKEEAENMMKKIDLLETSKRKLLGEGLGSCSIDELQKIEQQLER
SISKIRAKKTQVFREQIEQLKEKEKTLVAENAMLAEKYGNYSSQEATKDQRENIVEAETYADQSSPS
SDVETELFIGLPETRTRRISPKV

>Glyma09g40230=GmSOC1-a1
MVRGKTQLRRIENATSRQVTFSKRRNGLLKKAFELSVLCDAEVALIIFSPRGKLYEFASSSMQDTIE
RYRRHNRSAQTVNRSDEQNMQHLKQETANLMKKIELLEASKRKLLGEGLGSCSLEELQQIEQQLERS
VSSVRARKNQVYKEQIDQLKEKERALYAENARLCEQYGGIQPQPATKDPKEIQPYAESSPSSEVETE
LFIGLLRSSY

>Glyma18g45780=GmSOC1-a2
MVRGKTQMRRIENATSRQVTFSKRRNGLLKKAFELSVLCDAEVALIIFSPRGKLYEFASSSMQDTIE
RYRRHNRSAQTVNRSDEQNMQHLKQETANLMKKIELLEASKRKLLGEGLGSCSLEELQQIEQQLERS
VSNVRARKNQVYKEQIDQLKEKERALYAENARLCEQYGIQPQPATKDPKEIQPYAESSPSSEVETEL
FIGLPRSI

>Glyma10g38540=GmSOC1-a3
MVRGKVQLKKIEDTTSRQVTFSKRRSGLLKKAYELSVLCDAEVAVIVFSQNGRLYEFSSSDMTKILE
RYREHTKDVPASKFGDDYIQQLKLDSASLAKKIELLEHSKRELLGQSVSSCSYDELKGIEEQLQISL
QRVRQRKTQLYTEQIDQLRSQESNLLKENAKLSAMWQRAEKSSQQQWPRHTQAEAEPHCSSSQSLDV
DTELFIGLPKQQC

>Glyma20g29300=GmSOC1-a4
MARGKVQLKKIEDTTSRQVAFSKRRSGLLKKAYELSVLCDAEVAVIVFSQNGRLYEFSSSDMTKILE
RYREYTKDVPGSKFGDDYIQQLKLDSVSMTKKIELLEHSKRKLLGQSVSSCSFDELKGIEEQLRTSL
QRVRQRKTQLYTEQIDRLRSQESNLLKENAKLSAMYQRAERSSRQQWPRHTQAEAEPHCSSSQSLDV
DTELFIGLPKQQC

>Glyma17g14190=GmSOC1-a5
MVRGKTQMKRIENETSRQVTFSKRRNGLLKKAFELSVLCEAEVALIIFSTRGRLYEFSSSSVNKTVE
RYQRKIKDLGVSNKGIQKKTRHLKEGDMSMAKKIEHLEDSRRKLLGDELDKCSIDELQQLENQLERS
LDKIRARKNQLFRERIENLKQEEKCLLEVNKRLREQYRIDRQRCLTDNVTEKEAEEVETELFIGRPE
RRMPLKLKSATHSAHKYIA

>Glyma05g03660=GmSOC1-a6
MVRGKTQMKRIENETSRQVTFSKRRNGLLKKAFELSVLCDAEVALIIFSTRGRLYEFSSSRCSSINK
TVERYQRKIEDLGVSNKGIHENTQHLKEVDMSMAKKIEHLEDSRRKLLGDELDKCSIDELQQLENQL
ERSLDKIRATKNQLFRKRIEKLKEEEKCLLEVNKRLREQYRIERQRCLSDQDVEFATKKEGEEVETE
LFIGRPERRMPLKLKPTTYSAHAYIA

>Glyma07g08820=GmSOC1-b1
MVRGKTQMKRIENATSRQVTFSKRRNGLLKKAFELSVLCDAEVALIIFSPRGKLYEFASSSMQESIE
RYRRHTKHVNPTTFRSVEQNMQHLKQEAENMMKKIDLLEAAKRKFLGEGLGACSIEELQRIEQQLER
SLSNVRARKVQVFKEQIEQLKEKEKALLDENAKLTENARLSEKHDIHLQPATKNQNVNQPQCNAESS
SSSDVETELFIGLPDTRARHISHHV

>Glyma03g02200=GmSOC1-b2
MVRGKTQIKRIENATSRQVTFSKRRNGLLKKAFELSVLCDAEVALIIFSSSGKLYEFASSSIQCSIE
RYRRHTKHDNPTTFRSVEQNMQHLKQEAANMMKKIGLLEAAKRKFLGEGLGACSIEELQWIEQQLER
SLSNVRTRKIQVFKEQIEQLKGKHGINLQTETKNQNVNQPQYNAGSPSSDVETELIIGLPDTRTRRI
VHQLPQHQNGLEPNQRKEQPGVPKEELCNQQHEESH

>Ca_06279=CaSOC1-a
MVRGKTQLKRIENATSRQVTFSKRRNGLLKKAFELSVLCDAEVALIIFSPRGKLYEFSSSSMQDTIE
RYRRNSRSAQPMQKSDEQNMQNLRQETASLMKKIELLEASKRKLMGEGLGSCSMEELQQIEQQLEKS
VSTVRARKNQVYKNQIVQLKEKEKALLVENARLSKQGMQPQQTTKDQRENQPYAERSPSSDVVTELF
IGLHRS

>Ca_16423=CaSOC1-b
MVRGKTQMKRIENATSRQVTFSKRRNGLLKKAFELSVLCDAEVALIVFSPRGKLYEFASSSITGSIE
RYRSHTRTNNTSTPASVDQNTQAQLKEESQHMMKKIDLLEASKRKLLGQGLGSCSLDELQKIEQQLE
RSISSIRTKKNQIFREQIEQLKEKEKTLIAENIRLSEKYDKFSQPAKKDERENLHEPFAESSPRSDV
ETELFIGLPETRTWRISPKI

Table S3. Sequence of gene-specific primers and primers for reference genes used for qPCR analysis in the study.

RT-qPCR primers (Unique nucleotides are indicated in red)

LcSOC1-a (6g035350)
Fq: GTGTAAGCACTGTTCGAGCAAG 22bp, 50%GC, Tm=54.8C
Rq: CGCTTGATTATCTCTCGGACGA 22bp, 50%GC, Tm=54.8C
RevCom: TCGTCCGAGAGATAATCAAGCG
Amplicon size: 123 bp

LcSOC1-b1 (7g014230)
Fq: GATACCGCAGTCATACAAAGAATC 24bp, 42%GC, Tm=54C
Rq: TCTCCAACTGTTGCTCTACCC 21bp, 52%GC, Tm=54.4C
RevCom: GGGTAGAGCAACAGTTGGAGA
Amplicon size: 198 bp

LcSOC1-b2 (7g014090)
Fq: GGCTGAGGTTTTCAAAGAACAG 22bp, 45%GC, Tm=53C
Rq: GCATAAGCATCAACCTCATCTAC 23bp, 43%GC, Tm=53.5C
RevCom: GTAGATGAGGTTGATGCTTATGC
Amplicon size: 155 bp

LcSOC1-b3 (7g014020)
Fq: CTCAGGTTTACAGGGAGCAAATA 23bp, 43%GC, Tm=53.5C
Rq: GCTTGCTGTGATGAATAACTATCAC 25bp,40%GC,Tm=54.4C
RevCom: GTGATAGTTATTCATCACAGCAAGC
Amplicon size: 145 bp

LcSOC1-c1 (7g013900)
Fq: TGCTCTTATTGTTTACTCACCAAC 24bp, 38%GC,Tm=52.3C
Rq: ATCAATGGAACAACTCCCCAAG 22bp, 45%GC, Tm=53C
RevCom: CTTGGGGAGTTGTTCCATTGAT
Amplicon size: 241 bp

LcSOC1-c2 (7g014030)
Fq: ATTTTAGGAGAAGGTTTGGGGAG 23bp, 43%GC, Tm=53.5C
Rq: TCTCGACAAATCGCCCGAC 19bp, 58%GC, Tm=53.2C
RevCom: GTCGGGCGATTTGTCGAGA
Amplicon size: 175 bp

Lc-eIF4 (2g019770)
Fq: TCATCGGATTGGTCGTTCTGGA 22bp, 50%GC, Tm=54.8C
Rq: GGCATCTCATCAATCTGGGTAC 22bp, 50%GC, Tm=54.8C
RevCom: GTACCCAGATTGATGAGATGCC
Amplicon size: 126 bp

LcActin7 (L011470)
Fq: CTATGAGTTACCTGATGGACAG 22bp, 45%GC, Tm=53C
Rq: AACATAGTCGAACCACCACTCA 22bp, 45%GC, Tm=53C
RevCom: TGAGTGGTGGTTCGACTATGTT
Amplicon size: 201 bp

<u>Table S4.</u> Primers designed and used for Sanger sequences indicated in the genome fragment of the entire *LaSOC1-b2* (Lcu.2RBY.7g014090) gene in lentil.

<u>Primers</u>
LcSOC1a-F1: CTACTCTCAACTTCCCGGTGTT 22 bp, 50%GC, Tm=54.8C
LcSOC1a-R1: GGCCCTTTTAGATAAGTGGACG 22 bp, 50%GC, Tm=54.8C
(RevCom): CGTCCACTTATCTAAAAGGGCC
Amplicon size = 1,015 bp

LcSOC1a-F2: CGTCCACTTATCTAAAAGGGCC 22 bp, 50%GC, Tm=54.8C
LcSOC1a-R2: CTCCTCTCCAACTGTTGCTCTA 22 bp, 50%GC, Tm=54.8C
(RevCom): TAGAGCAACAGTTGGAGAGGAG
Amplicon size = 891 bp

<u>Lcu.2RBY.7g014090</u>
ATGGTGAGAGGGAAGACACAGATGAAGCGTATAGAGAACGCAACAAGCAGACAAGTAACATTTTCAA
AGAGAAGAAATGGTTTGATGAAGAAAGCTTTTGAGTTATCAGTTTTGTGTGATGCTGAAGTTGCTCT
TATTGTTTTTTCATCAAGAGGGAGACTTTATGAATTTGCAAGCTCAGGGTATGTATGTCATGTACTT
TTTTTAGATTTAATATTGTTTTCAATATTTAAGTGTGCTAAAAGAGTTTATAACTTGTCGGTAAATA
TTACTCCTAACTCAACGGTAAAAAAAAAATATAGTGGTAAACCGATAGATGTCAATACTAGAGTTTA
AATCCTGAATCTGATGATTAATCTCATGGTTGATTACATAAATTTATAGTAATTATCACCACTTCGT
CTATCAATAAAAAACAAAGTCTTTTAATGTTTTTTCTAGTTTAAGGAAGCTTTAGGTTTTTTCTATG
TTACACTGGAACATAGATTTTTCAAATTTAAATCTGTGAACTAAAATTTATAACAATACTAATCCTT
ATTGTTCTCAGAAATAATTCACGCGTAGAAATCACAATGTATTCAACATCAATTTAAAAAAATGTAT
TTAAAGCTACTAATTTTAAACTAAATCCATGACCAAAATAAAAAATTAACATAAAATATAATGACAC
CAAGCAACACAATATATAATCAAAACTATCATATGTTAGAAAAAACTCATGTGTAGAAATCACAATG
TATTTAACAACATCAATAAAAAAAGATGTATCTAGATCTACAAATTTTAACATAAATCCATGAAATA
AAATAAAAAACATAACAAAATATATAATCAAAACTATCATATGTTAGAAAATCATTTTAATTCCATA
AGTAGTTTAATGCTTAATCTCACCCATAAATCAAGATTTGATTTTGCTCCTCCAACATCTCATATGA
TTAATAATAAAAAATATCATAATTTGATTTATCTTAAGGGTGGTATATGCTAGTACTTTTCTTTTAT
AATTACTAATATTAATGTGAATTATTAATAAAAAAAGAAAATGCAGTGAGGTCACAACATTGTTTAG
CATATAAATAACACTTCAGGTCACCAAAACATTAACATCCCAAGGCTGTTAAGTTATATACACGTAT
TTGTTATCATTAATATGACATCCAACTTAAGCTTCTATGATCATTATCTTTTGTTGTTGTCTCTTGT
TTTGTTGGGTATGTGTATTAGTTACTGTAACCGTTTATTCTTTGTATTAATTGTTTGATAGAATGTG
ATGTTTATGTTATTCATTAATATCTCTTAAGCTTTACATAGTCATAGCTGTTAAATTTTAGTTAATT
AGTAACATGAATAGTTAATTTTCTGATATATTTTGTTTATGTCAATTTTTGTGTTTTCTAGTGGTTA
TAAGCATAACTTACATCCTCTCATCTTTTTTATTCTCTATTATTTATAGAGAAATATCGTAAGTTCT
ATTATAAGAAATAAAACATTAATAAAATTCAAATTGCAAAATTAGTCTTTCTTCTATATTTTTCTCT
ACCAAATTTTTGTTGTCCCAACAAATTTGATACTAGAGTTTTGGCCCATTACGTAGCATGATTGCAT
CAAATAGGAATTCTATTTTTTCATATACAACAAGCAAAATTTCAATTTTCGATGGTGAGCACTATGA
TTTCTGGAATTCACAAATGGAGACGATCTATTGGGCGTGGTTGAAGAAGGTTATGACTTATGAAGAT
CGTCCACAACCAAAATCAGTAGATTGGTCTGATGATAAAGCGAAATAATACAAAGAGAATGTAAAGA
ACAATGATACAACATTATGATTTGGCTTTAATTTATTGGGCAAAAATCTTAGGTTTTCACTATGTTC
TTTCACTTCAAAGCTTTTACTGAGAAGCAGGGTGGCTATGAGATAACTACTCTTAGAACAGATCGAG
GAGGCGAATTTATCCATAAGCTATTTCTCAACTATTGCAAGGAACAAGACATTGAGAGGAAGCTTAC
CATCAGATACACACCACAACAGAACGGTGTGGCAGAAAGGAAAAATCGCACAATTGTTGAAATGGAA
AGGAGCGTGTTAAAAGGAATTAAACTTCCAAACAAATTATGGTTGAAGCTGTTAATAGTGCTCCATA

CATTCTAAATAGATCTCCTACAAAGGCAGTCCAAAACAAAACACCATATGAACCATGGCATGGAAGA
AAGCCAATTGTAAGTCATTTTAAAGTATTTGGTTGTATTGCTTATTCTATCATTCCTTCCCAAAAGT
GATAAAAGTTTGATGAAAAAAGTGAAAAACTCATTTTCGTTGGATTTAGTGATGAGTCAAAAGGTTA
TCATCTTTATAATCAAATGACTAATCAAATGGTACTTTCAAGAGATGTAATTTTTTGTGAAAGCACA
TCATGAAAATGGGAATATGAAGATGTTGATCATGGGTTTGTTGAGTTTGCGCCTCTTGCACCGCTTA
AGCAATCTCCTTCAAATGTTAGTGGCCTATCAAGGAGTGATAATAATGAAGATGATTTAGATTTAGA
AACTCGTCCAAGAAAGTTTCGTTCTTTATCTGAAATTTATGAATCATGCAATGTTGTCTTTTTTGCA
AGTGAACGACAATGCTTTAAGGAATTTGTTGAAGGACATGTGTGGAGAAAAGCTTTGAATGAAGAAA
TAAAGAGTATTGAGAAAAAAATCAAACTTGGGATCTTGTGGATCTTCCATAAGGAAAACATTCAATT
GGTTTGAAATGGGTGTATAAGACCAAATACAACGAAAATGGTTGTGTGCAAAAATACAAAGCAAGAT
TAGTAGTAAAAGGCTACTCTCAACTTCCCGGTGTTGATTTTAATGAGACATTTTCTCATGTTGCACG
CATGGAGACAATCAGAACAATACTTGCCATGGCAGCTCAGATGGAGTTGCAAGTTTTTTAGTTTGAT
GTCAAATCAGTCTTCCTTAACGGAGAATTAGAAGAAGAAGACTAAGTTCAACAGCCAAAAGGAATTG
GGAAAGAAGGAAAGTTTTCAAATTGAAGAAATCACTTTATGGCCTCAAACAAACGCCAAAAGCATGA
AATAACAAGATTGATGCTTATTTTTGGCAAAGTGGATTTTTGAGAAGTCCATCTGAACCATCTTTCT
ATGTGAAGAGAAATGGAGAAGATTTTCTAATGGTTTGTCTTTATATTGTTGATTTAATTTATGTAGG
GACTGATATTAATATGGTGGACTATTTTAAGAAAGCAATGAAGAAGGAATATGAAATGACAAATCTT
AGATTGATGAGATATTTCCTTGGAATTCAAGTAAAGCAAATAAAAGGGGAGATTTTTATCACTAGTA
CGTTTTAGATATGTTTAAAAATTCAGAATGGAGAATTGTAAACCAGCGTCATAGAATTGTAAACTAG
AATGAAGAAGTACGTTTTAAATATGCTTGAAAAATTCAAAATGGATTGTAAACCAACATCGTAGAAT
TGTAAACTAGAATGGAGAAGTATGTTTTCCCATATGATACTCATTCTGATCATAGATAGAAAAAAGC
ATAGGAAATCTAACTTTAAGAGAAGTGACCCTCATCTAAGGATCACTCTAGGAAGAAGTGAGTAGAC
CTTATCTAACCTGTTTAAGCAAACCGTCTCGAAACTAGTCAGAATGGTCCTCCACTATAACCCCTAC
AAGAGACACCCCTCTCAATCAAGGAGACACATATAGAAGACCCCAAACTCTACTATCCAAAATAGAC
AAAATCGACGTACCCCATGTCTAGCGGATAAAATATCACACCAAATAAACACTTGAAAGCAACCATT
ACGTCCACTTATCTAAAAGGGCCAGGTTCACCTTCCGCAGGTCGAACTTCTTAGGATTAAACAAGAT
CTAACATATATATATATATATATATATATATATATATATATATATATATATATATATATTATACATATA
TATTATATACCTTATTATGAATGAGAGTAAAATGAAGAAATTGTAATTTCCCTAATTAATTACAATA
GTATACTGCTTATTTATGCTAATACAAAGTGTGTAAGATTTATTCATCTTTTCCTTCTCTATACATT
TGCCAAAAATTTGATTATCTGTGTAGTGCATATGAAACTAAAGAAAGGCAATGAGTGAGTTTTTTAC
TTTTCAATTTTCCCTCCCTTTTACATATCCTATTGATATTTCATTTTCTCATATGTATCCTTCAGAT
TTTTCTCATTTTCTAACCAATTATTAGCAAGTGCCTTATTATATATATCTGGACCTTTAATTAATCA
GTAACATTCCCTTTATTGTTACAGCATTCTAGAAACAATTGAACGATACCGCAGTCATAGCCGGATT
AATAATAATTCAACAACAACCGAATCTGCTGAAAATACTCAGGTTAATTTAATTACATATACTGTCT
TTTTTCACTCTCATTTTCAACATGATTAGAGACTTGAAGTTTGAATGAAACTGTCATATATTACAGC
ATTTGAAGGAAGAAGCAGAGAACATGATGAAAAAGATTGATCTTCTTGAGACTTCTAAACGGTTAAA
CATCTTGAATTTATAATCATGCTGACACATATTTTTACAAGATTCAAAGTCGAAGATGAAAATATAT
TCTGCGTTTGCAGAAAACTCTTAGGAGAAGGTTTAGGAACTTGTTCCATTGATGAACTGCAAAGGAT
AGAGCAACAGTTGGAGAGGAGTATAACCAAAATTCGAGTTAGAAAGGTTAGAATTTTCAAAAATCTG
GTGAACTATAAAAATATAAAGAAATTATTTTACTGATGAACTTTTTATTTTTCAGGCTGAGGTTTTC
AAAGAACAGATTGATCAGCTAAAAGAAAAGGTAAGCTGTTGAAAAAAACTCCAACCTATAATTACAT
AAAAAAGTTTCTAATGTGAAGAAAAATAATTTGAATACAATTCAGGAAAAAACCCTAGTTGCTGAAA
ATACCAGGCTCTCTGAGAAGGTGAGTTCACTTTCTATTTGAAATTAATTCTAGCCCTAACTAATAAA
AATATTTGATAACAAATTCAAAACATTCTTCTCTAGTTTTACAAAACCATTGTTGTTATGGAATGAA
AATCCAAATATTTTGTGCAGTATAATAACTATTCATCACAGCAAGGAAAAAAGGATGATAGAGTAAA
TGTAGATGAGGTTGATGCTTATGCAGATCAAAGTAGTCCAAATTCAGATGTTGAAACTGAGTTATTC
ATTGGTCTTCCAGAAACAAGAACAAGGAAAATTTCTCCAATGTTGAGGACTAACTAA

Table S5. ASQ genotyping for *LcSOC1-b2*: PCR cocktail composition, sequences of the allele specific (AS) primers and universal (Uni) molecular probes

ASQ PCR cocktail composition

	Component	For one reaction (µl)	Final concentration
1	DNA template (20 ng/µl)	2.0	4 ng/µl
2	10×SE Buffer (including1.5 mM $MgCl_2$)	1.0	1×
3	dNTP (2 mM)	1.0	0.2 mM
4	AS-primer mix (F1 = 1 µM; F2 = 1 µM; and R = 5 µM)	1.0	0.1+0.1+ +0.5 µM
5	Uni-probe mix (Uni-1 = 1 µM; Uni-2 = 1 µM; and Uni-Q = 10 µM)	0.3	0.03+0.03+ +0.3 µM
6	$MgCl_2$ (25 mM) additional	0.1	1.5+0.25= =1.75 mM
7	SibEnzyme Taq polymerase (5 U/µl)	0.04	0.02 U/µl
8	Water	4.56	
	Total	10.0	

Note: Passive dye ROX was added in Master-mix (1-3 µl) for a few samples or entire microplate when Thermo Fisher qPCR instrument was used. ROX is not required for other qPCR machines, like BioRad.

Amplification program

(1) 95C – 1 min – Initial denaturation
(2) 95C – 10 sec
(3) 59C – 30 sec
(4) Go to step 2 for 10 cycles more – First round of cycles
(5) 95C – 10 sec
(6) 60C – 10 sec
(7) 69C – 45 sec
(8) 54C – 50 sec + Plate read
(9) Go to step 5 for 25 cycles more – Second round of cycles

Note: The duration of the entire program is approximately 1 h and 30 min.

***LcSOC1-b2*:**

SNP1

TTTAATGAGACATTTTCTCATGTTGCACGCATGGAGACAATCAGAACAATACTTGCCATGGCAGCTC
AGATGGAGTTGCAAGTTTTTTAGTTTGATG**[T/C]**CAAATCAGTCTTCCTTAACGGAGAATTAGAA
GAAGAAGACTAAGTTCAACAGCCAAAAGGAATTGGGAAAGAAGGAAAGTTTTCAAATTGAAGAAATC

SNP1-F1: TCCGTTAAGGAAGACTGATTTG**A**C 24bp, 42%GC, Tm=54C
(RevCom): G**T**CAAATCAGTCTTCCTTAACGGA

SNP1-F2: CCGTTAAGGAAGACTGATTTG**G**C 23bp, 48%GC, Tm=55.3C
(RevCom): G**C**CAAATCAGTCTTCCTTAACGG

SNP1-R: TGTTGCACGCATGGAGACAATCA, 23bp, 48%GC, Tm=55.3C
Amplicon size: 99 bp

SNP2

AAATGGATTGTAAACCAACATCGTAGAATTGTAAACTAGAATGGAGAAGTATGTTTTCCCATATGAT
ACTCATTCTGATCATAGATAGAAAAAA**[G/A]**CATAGGAAATCTAACTTTAAGAGAAGTGACCCTC

SNP2-F1: CTTCTCTTAAAGTTAGATTTCCTATG**C**T 28bp, 32%GC, Tm=54C
(RevCom): A**G**CATAGGAAATCTAACTTTAAGAGAAG

SNP2-F2: CTTCTCTTAAAGTTAGATTTCCTATG**T**T 28bp, 29%GC, Tm=52.6C
(RevCom): A**A**CATAGGAAATCTAACTTTAAGAGAAG

SNP2-R: CCAACATCGTAGAATTGTAAACTAGA 26bp, 35%GC, Tm=53.2C
Amplicon size: 107 bp

Allele-specific primers with tags and tails (5'-3')

LcSOC1b2-SNP1-F1: GTCCTTGCGAAGGCATCCTCCGTTAAGGAAGACTGATTTG**A**C
LcSOC1b2-SNP1-F2: GTCCTTGCGAAGGCCAACCCGTTAAGGAAGACTGATTTG**G**C
LcSOC1b2-SNP1-R: TGTTGCACGCATGGAGACAATCA

LcSOC1b2-SNP2-F1: GTCCTTGCGAAGGCATCCCTTCTCTTAAAGTTAGATTTCCTATG**C**T
LcSOC1b2-SNP2-F2: GTCCTTGCGAAGGCCAACCTTCTCTTAAAGTTAGATTTCCTATG**T**T
LcSOC1b2-SNP2-R: CCAACATCGTAGAATTGTAAACTAGA

Universal ASQ probes (5'-3')

Uni1-FAM: FAM-GTCCTTGCGAAGGCATCC
Uni2-VIC: VIC-GTCCTTGCGAAGGCCAAC
Uni-Q: GCCTTCGCAAGGAC-**BHQ1**

<u>Table S6</u>. Genome sequence of *LcSOC1-b2* gene with the two identified SNP indicated in the studied lentil accessions.

>Lcu.2RBY.7g014090. lencu.CDC_Redberry.gnm2.ann1. Lcu.2RBY. Chr7:60463873..60469848 (+ strand) class=mRNA length=5976
MADS-box transcription factor 6 [Glycine max]; IPR002100 (Transcription factor, MADS-box), IPR002487 (Transcription factor, K-box); GO:0003677 (DNA binding), GO:0003700 (sequence-specific DNA binding transcription factor activity), GO:0005634 (nucleus), GO:0046983 (protein dimerization activity). legfed_v1_0.L_6RNLR0. Lens culinaris. CDC_Redberry

ATG – 'Start' codon; TAA – 'Stop' codon. Yellow – Exons; and no colours – Introns or UTRs (Untranslated regions).

CTTTTGGGGATGCATAGTTTCTTCTTTCTCTTGATTCCTTAGCTCTTATTTATTTTTATCCTTCTTT
TCCTTTTTTAGACTAAAACCTTTATTCTTATCTCTTCTCTCCTTCATTTTCTTCTCTATTTTGGTGC
AAAGCTGTTTCCTCTTGAAGTTGCTTTTCTTGCTGGTAAGTTCAGAAAAATCACTTATTTCATATGA
GATATAAATCTTTAACCTACATGTTTATAATAGACCCTTATACTACAACCTACTTTACACCATTTAA
TTTTCTCATCAAAATACTTTGACAATTATCTCACTCTTTTACTTGAAAACTTTATGATATAACTTTA
TTTTATATTTTCATTAAACTTCCTATTTATTTCAGTCTCAAAATTCAATTCAACCTTAAGATTTTGT
TTTCATTGTTTGGTAAGTGATTCTAGTAAGTCTCTTGCATTTTTTTTCGTCTCTCACAGTTTTTCTA
GATCTTAAAAGCTATTATACTTTCATCCCCTTTTACTTTTTTGGTTGGTAATTTGTGCTTTAATTCC
TTCTTGGTGAGGATTGTCTTGTTATATATTGACCATGAAATTCAATTTTTTTTTCATATTTTTTTGT
TGATGTTGTGATTTTCAGCAAAGATGGTGAGAGGGAAGACACAGATGAAGCGTATAGAGAACGCAAC
AAGCAGACAAGTAACATTTTCAAAGAGAAGAAATGGTTTGATGAAGAAAGCTTTTGAGTTATCAGTT
TTGTGTGATGCTGAAGTTGCTCTTATTGTTTTTTCATCAAGAGGGAGACTTTATGAATTTGCAAGCT
CAGGGTATGTATGTCATGTACTTTTTTTAGATTTAATATTGTTTTCAATATTTAAGTGTGCTAAAAG
AGTTTATAACTTGTCGGTAAATATTACTCCTAACTCAACGGTAAAAAAAAAAATATAGTGGTAAACCG
ATAGATGTCAATACTAGAGTTTAAATCCTGAATCTGATGATTAATCTCATGGTTGATTACATAAATT
TATAGTAATTATCACCACTTCGTCTATCAATAAAAAACAAAGTCTTTTAATGTTTTTTCTAGTTTAA
GGAAGCTTTAGGTTTTTTCTATGTTACACTGGAACATAGATTTTTCAAATTTAAATCTGTGAACTAA
AATTTATAACAATACTAATCCTTATTGTTCTCAGAAATAATTCACGCGTAGAAATCACAATGTATTC
AACATCAATTTAAAAAAATGTATTTAAAGCTACTAATTTTAAACTAAATCCATGACCAAAATAAAAA
ATTAACATAAAATATAATGACACCAAGCAACACAATATATAATCAAAACTATCATATGTTAGAAAAA
ACTCATGTGTAGAAATCACAATGTATTTAACAACATCAATAAAAAAAGATGTATCTAGATCTACAAA
TTTTAACATAAATCCATGAAATAAAATAAAAAACATAACAAAATATATAATCAAAACTATCATATGT
TAGAAAATCATTTTAATTCCATAAGTAGTTTAATGCTTAATCTCACCCATAAATCAAGATTTGATTT
TGCTCCTCCAACATCTCATATGATTAATAATAAAAAATATCATAATTTGATTTATCTTAAGGGTGGT
ATATGCTAGTACTTTTCTTTTATAATTACTAATATTAATGTGAATTATTAATAAAAAAAGAAAATGC
AGTGAGGTCACAACATTGTTTAGCATATAAATAACACTTCAGGTCACCAAAACATTAACATCCCAAG
GCTGTTAAGTTATATACACGTATTTGTTATCATTAATATGACATCCAACTTAAGCTTCTATGATCAT
TATCTTTTGTTGTTGTCTCTTGTTTTGTTGGGTATGTGTATTAGTTACTGTAACCGTTTATTCTTTG
TATTAATTGTTTGATAGAATGTGATGTTTATGTTATTCATTAATATCTCTTAAGCTTTACATAGTCA
TAGCTGTTAAATTTTAGTTAATTAGTAACATGAATAGTTAATTTTCTGATATATTTTGTTTATGTCA
ATTTTTGTGTTTTCTAGTGGTTATAAGCATAACTTACATCCTCTCATCTTTTTTATTCTCTATTATT

TATAGAGAAATATCGTAAGTTCTATTATAAGAAATAAAACATTAATAAAATTCAAATTGCAAAATTA
GTCTTTCTTCTATATTTTTCTCTACCAAATTTTTGTTGTCCCAACAAATTTGATACTAGAGTTTTGG
CCCATTACGTAGCATGATTGCATCAAATAGGAATTCTATTTTTTCATATACAACAAGCAAAATTTCA
ATTTTCGATGGTGAGCACTATGATTTCTGGAATTCACAAATGGAGACGATCTATTGGGCGTGGTTGA
AGAAGGTTATGACTTATGAAGATCGTCCACAACCAAAATCAGTAGATTGGTCTGATGATAAAGCGAA
ATAATACAAAGAGAATGTAAAGAACAATGATACAACATTATGATTTGGCTTTAATTTATTGGGCAAA
AATCTTAGGTTTTCACTATGTTCTTTCACTTCAAAGCTTTTACTGAGAAGCAGGGTGGCTATGAGAT
AACTACTCTTAGAACAGATCGAGGAGGCGAATTTATCCATAAGCTATTTCTCAACTATTGCAAGGAA
CAAGACATTGAGAGGAAGCTTACCATCAGATACACACCACAACAGAACGGTGTGGCAGAAAGGAAAA
ATCGCACAATTGTTGAAATGGAAAGGAGCGTGTTAAAAGGAATTAAACTTCCAAACAAATTATGGTT
GAAGCTGTTAATAGTGCTCCATACATTCTAAATAGATCTCCTACAAAGGCAGTCCAAAACAAAACAC
CATATGAACCATGGCATGGAAGAAAGCCAATTGTAAGTCATTTTAAAGTATTTGGTTGTATTGCTTA
TTCTATCATTCCTTCCCAAAAGTGATAAAAGTTTGATGAAAAAAGTGAAAAACTCATTTTCGTTGGA
TTTAGTGATGAGTCAAAAGGTTATCATCTTTATAATCAAATGACTAATCAAATGGTACTTTCAAGAG
ATGTAATTTTTTGTGAAAGCACATCATGAAAATGGGAATATGAAGATGTTGATCATGGGTTTGTTGA
GTTTGCGCCTCTTGCACCGCTTAAGCAATCTCCTTCAAATGTTAGTGGCCTATCAAGGAGTGATAAT
AATGAAGATGATTTAGATTTAGAAACTCGTCCAAGAAAGTTTCGTTCTTTATCTGAAATTTATGAAT
CATGCAATGTTGTCTTTTTTGCAAGTGAACGACAATGCTTTAAGGAATTTGTTGAAGGACATGTGTG
GAGAAAAGCTTTGAATGAAGAAATAAAGAGTATTGAGAAAAAAATCAAACTTGGGATCTTGTGGATC
TTCCATAAGGAAAACATTCAATTGGTTTGAAATGGGTGTATAAGACCAAATACAACGAAAATGGTTG
TGTGCAAAAATACAAAGCAAGATTAGTAGTAAAAGGCTACTCTCAACTTCCCGGTGTTGATTTTAAT
GAGACATTTTCTCATGTTGCACGCATGGAGACAATCAGAACAATACTTGCCATGGCAGCTCAGATGG
AGTTGCAAGTTTTTTAGTTTGATG **[T/C]** CAAATCAGTCTTCCTTAACGGAGAATTAGAAGAAGAA
GACTAAGTTCAACAGCCAAAAGGAATTGGGAAAGAAGGAAAGTTTTCAAATTGAAGAAATCACTTTA
TGGCCTCAAACAAACGCCAAAAGCATGAAATAACAAGATTGATGCTTATTTTTGGCAAAGTGGATTT
TTGAGAAGTCCATCTGAACCATCTTTCTATGTGAAGAGAAATGGAGAAGATTTTCTAATGGTTTGTC
TTTATATTGTTGATTTAATTTATGTAGGGACTGATATTAATATGGTGGACTATTTTAAGAAAGCAAT
GAAGAAGGAATATGAAATGACAAATCTTAGATTGATGAGATATTTCCTTGGAATTCAAGTAAAGCAA
ATAAAAGGGGAGATTTTTATCACTAGTACGTTTTAGATATGTTTAAAAATTCAGAATGGAGAATTGT
AAACCAGCGTCATAGAATTGTAAACTAGAATGAAGAAGTACGTTTTAAATATGCTTGAAAAATTCAA
AATGGATTGTAAACCAACATCGTAGAATTGTAAACTAGAATGGAGAAGTATGTTTTCCCATATGATA
CTCATTCTGATCATAGATAGAAAAAA **[G/A]** CATAGGAAATCTAACTTTAAGAGAAGTGACCCTCA
TCTAAGGATCACTCTAGGAAGAAGTGAGTAGACCTTATCTAACCTGTTTAAGCAAACCGTCTCGAAA
CTAGTCAGAATGGTCCTCCACTATAACCCCTACAAGAGACACCCCTCTCAATCAAGGAGACACATAT
AGAAGACCCCAAACTCTACTATCCAAAATAGACAAAATCGACGTACCCCATGTCTAGCGGATAAAAT
ATCACACCAAATAAACACTTGAAAGCAACCATTACGTCCACTTATCTAAAAGGGCCAGGTTCACCTT
CCGCAGGTCGAACTTCTTAGGATTAAACAAGATCTAACATATATATATATATATATATATATATATA
TATATATATATATATATATATATTATACATATATATTATATACCTTATTATGAATGAGAGTAAAATG
AAGAAATTGTAATTTCCCTAATTAATTACAATAGTATACTGCTTATTTATGCTAATACAAAGTGTGT
AAGATTTATTCATCTTTTCCTTCTCTATACATTTGCCAAAAATTTGATTATCTGTGTAGTGCATATG
AAACTAAAGAAAGGCAATGAGTGAGTTTTTTACTTTTCAATTTTCCCTCCCTTTTACATATCCTATT
GATATTTCATTTTCTCATATGTATCCTTCAGATTTTTCTCATTTTCTAACCAATTATTAGCAAGTGC
CTTATTATATATATCTGGACCTTTAATTAATCAGTAACATTCCCTTTATTGTTACAGCATTCTAGAA
ACAATTGAACGATACCGCAGTCATAGCCGGATTAATAATAATTCAACAACAACCGAATCTGCTGAAA
ATACTCAGGTTAATTTAATTACATATACTGTCTTTTTTCACTCTCATTTTCAACATGATTAGAGACT
TGAAGTTTGAATGAAACTGTCATATATTACAGCATTTGAAGGAAGAAGCAGAGAACATGATGAAAAA
GATTGATCTTCTTGAGACTTCTAAACGGTTAAACATCTTGAATTTATAATCATGCTGACACATATTT
TTACAAGATTCAAAGTCGAAGATGAAAATATATTCTGCGTTTGCAGAAAACTCTTAGGAGAAGGTTT

AGGAACTTGTTCCATTGATGAACTGCAAAGGATAGAGCAACAGTTGGAGAGGAGTATAACCAAAATT
CGAGTTAGAAAGGTTAGAATTTTCAAAAATCTGGTGAACTATAAAAATATAAAGAAATTATTTTACT
GATGAACTTTTTATTTTTCAGGCTGAGGTTTTCAAAGAACAGATTGATCAGCTAAAAGAAAAGGTAA
GCTGTTGAAAAAAACTCCAACCTATAATTACATAAAAAAGTTTCTAATGTGAAGAAAAATAATTTGA
ATACAATTCAGGAAAAAACCCTAGTTGCTGAAAATACCAGGCTCTCTGAGAAGGTGAGTTCACTTTC
TATTTGAAATTAATTCTAGCCCTAACTAATAAAAATATTTGATAACAAATTCAAAACATTCTTCTCT
AGTTTTACAAAACCATTGTTGTTATGGAATGAAAATCCAAATATTTTGTGCAGTATAATAACTATTC
ATCACAGCAAGGAAAAAAGGATGATAGAGTAAATGTAGATGAGGTTGATGCTTATGCAGATCAAAGT
AGTCCAAATTCAGATGTTGAAACTGAGTTATTCATTGGTCTTCCAGAAACAAGAACAAGGAAAATTT
CTCCAATGTTGAGGACTAACTAATCACTTTAACTATGTAAAATGAAAATAAGGATAAGGCAAAGTGT
CTCATTAATATTTTATATTGGAT

<u>Table S7</u>. Haplotypes of the *LcSOC1-b2* gene based on SNP1 and SNP2 in eight studied lentil accessions.

```
Y705-LebaneseLocal      ATGTTGCACGCATGGGAGACAATCAGAACAATACTTGCCATGGCAGCTCAGATGGAGTTG
Y706-SyrianLocal        ATGTTGCACGCATGGGAGACAATCAGAACAATACTTGCCATGGCAGCTCAGATGGAGTTG
Y708-Niva-95            ATGTTGCACGCATGGGAGACAATCAGAACAATACTTGCCATGGCAGCTCAGATGGAGTTG
Y704-Flip92-36L         ATGTTGCACGCATGGGAGACAATCAGAACAATACTTGCCATGGCAGCTCAGATGGAGTTG
Y703-Krapinka           ATGTTGCACGCATGGGAGACAATCAGAACAATACTTGCCATGGCAGCTCAGATGGAGTTG
Y701-Flip96-48L         ATGTTGCACGCATGGGAGACAATCAGAACAATACTTGCCATGGCAGCTCAGATGGAGTTG
Y702-ILL-1552           ATGTTGCACGCATGGGAGACAATCAGAACAATACTTGCCATGGCAGCTCAGATGGAGTTG
Y707-Vekhovskaya-1      ATGTTGCACGCATGGGAGACAATCAGAACAATACTTGCCATGGCAGCTCAGATGGAGTTG
                        ************************************************************
                                          SNP-1
Y705-LebaneseLocal      CAAGTTTTTTAGTTTGATGCCAAATCAGTCTTCCTTAACGGAGAATTAGAAGAAGAAGAC
Y706-SyrianLocal        CAAGTTTTTTAGTTTGATGCCAAATCAGTCTTCCTTAACGGAGAATTAGAAGAAGAAGAC
Y708-Niva-95            CAAGTTTTTTAGTTTGATGCCAAATCAGTCTTCCTTAACGGAGAATTAGAAGAAGAAGAC
Y704-Flip92-36L         CAAGTTTTTTAGTTTGATGCCAAATCAGTCTTCCTTAACGGAGAATTAGAAGAAGAAGAC
Y703-Krapinka           CAAGTTTTTTAGTTTGATGCCAAATCAGTCTTCCTTAACGGAGAATTAGAAGAAGAAGAC
Y701-Flip96-48L         CAAGTTTTTTAGTTTGATGCCAAATCAGTCTTCCTTAACGGAGAATTAGAAGAAGAAGAC
Y702-ILL-1552           CAAGTTTTTTAGTTTGATGTCAAATCAGTCTTCCTTAACGGAGAATTAGAAGAAGAAGAC
Y707-Vekhovskaya-1      CAAGTTTTTTAGTTTGATGTCAAATCAGTCTTCCTTAACGGAGAATTAGAAGAAGAAGAC
                        ******************* ****************************************

Y705-LebaneseLocal      TAAGTTCAACAGCCAAAAGGAATTGGGAAAGAAGGAAAGTTTTCAAATTGAAGAAATCAC
Y706-SyrianLocal        TAAGTTCAACAGCCAAAAGGAATTGGGAAAGAAGGAAAGTTTTCAAATTGAAGAAATCAC
Y708-Niva-95            TAAGTTCAACAGCCAAAAGGAATTGGGAAAGAAGGAAAGTTTTCAAATTGAAGAAATCAC
Y704-Flip92-36L         TAAGTTCAACAGCCAAAAGGAATTGGGAAAGAAGGAAAGTTTTCAAATTGAAGAAATCAC
Y703-Krapinka           TAAGTTCAACAGCCAAAAGGAATTGGGAAAGAAGGAAAGTTTTCAAATTGAAGAAATCAC
Y701-Flip96-48L         TAAGTTCAACAGCCAAAAGGAATTGGGAAAGAAGGAAAGTTTTCAAATTGAAGAAATCAC
Y702-ILL-1552           TAAGTTCAACAGCCAAAAGGAATTGGGAAAGAAGGAAAGTTTTCAAATTGAAGAAATCAC
Y707-Vekhovskaya-1      TAAGTTCAACAGCCAAAAGGAATTGGGAAAGAAGGAAAGTTTTCAAATTGAAGAAATCAC
                        ************************************************************

Y705-LebaneseLocal      TTTATGGCCTCAAACAAACGCCAAAAGCATGAAATAACAAGATTGATGCTTATTTTTGGC
Y706-SyrianLocal        TTTATGGCCTCAAACAAACGCCAAAAGCATGAAATAACAAGATTGATGCTTATTTTTGGC
Y708-Niva-95            TTTATGGCCTCAAACAAACGCCAAAAGCATGAAATAACAAGATTGATGCTTATTTTTGGC
Y704-Flip92-36L         TTTATGGCCTCAAACAAACGCCAAAAGCATGAAATAACAAGATTGATGCTTATTTTTGGC
Y703-Krapinka           TTTATGGCCTCAAACAAACGCCAAAAGCATGAAATAACAAGATTGATGCTTATTTTTGGC
Y701-Flip96-48L         TTTATGGCCTCAAACAAACGCCAAAAGCATGAAATAACAAGATTGATGCTTATTTTTGGC
Y702-ILL-1552           TTTATGGCCTCAAACAAACGCCAAAAGCATGAAATAACAAGATTGATGCTTATTTTTGGC
Y707-Vekhovskaya-1      TTTATGGCCTCAAACAAACGCCAAAAGCATGAAATAACAAGATTGATGCTTATTTTTGGC
                        ************************************************************

Y705-LebaneseLocal      AAAGTGGATTTTTGAGAAGTCCATCTGAACCATCTTTCTATGTGAAGAGAAATGGAGAAG
Y706-SyrianLocal        AAAGTGGATTTTTGAGAAGTCCATCTGAACCATCTTTCTATGTGAAGAGAAATGGAGAAG
Y708-Niva-95            AAAGTGGATTTTTGAGAAGTCCATCTGAACCATCTTTCTATGTGAAGAGAAATGGAGAAG
Y704-Flip92-36L         AAAGTGGATTTTTGAGAAGTCCATCTGAACCATCTTTCTATGTGAAGAGAAATGGAGAAG
Y703-Krapinka           AAAGTGGATTTTTGAGAAGTCCATCTGAACCATCTTTCTATGTGAAGAGAAATGGAGAAG
Y701-Flip96-48L         AAAGTGGATTTTTGAGAAGTCCATCTGAACCATCTTTCTATGTGAAGAGAAATGGAGAAG
Y702-ILL-1552           AAAGTGGATTTTTGAGAAGTCCATCTGAACCATCTTTCTATGTGAAGAGAAATGGAGAAG
Y707-Vekhovskaya-1      AAAGTGGATTTTTGAGAAGTCCATCTGAACCATCTTTCTATGTGAAGAGAAATGGAGAAG
                        ************************************************************

Y705-LebaneseLocal      ATTTTCTAATGGTTTGTCTTTATATTGTTGATTTAATTTATGTAGGGACTGATATTAATA
Y706-SyrianLocal        ATTTTCTAATGGTTTGTCTTTATATTGTTGATTTAATTTATGTAGGGACTGATATTAATA
Y708-Niva-95            ATTTTCTAATGGTTTGTCTTTATATTGTTGATTTAATTTATGTAGGGACTGATATTAATA
Y704-Flip92-36L         ATTTTCTAATGGTTTGTCTTTATATTGTTGATTTAATTTATGTAGGGACTGATATTAATA
Y703-Krapinka           ATTTTCTAATGGTTTGTCTTTATATTGTTGATTTAATTTATGTAGGGACTGATATTAATA
Y701-Flip96-48L         ATTTTCTAATGGTTTGTCTTTATATTGTTGATTTAATTTATGTAGGGACTGATATTAATA
Y702-ILL-1552           ATTTTCTAATGGTTTGTCTTTATATTGTTGATTTAATTTATGTAGGGACTGATATTAATA
Y707-Vekhovskaya-1      ATTTTCTAATGGTTTGTCTTTATATTGTTGATTTAATTTATGTAGGGACTGATATTAATA
                        ************************************************************
```

```
Y705-LebaneseLocal        TGGTGGACTATTTTAAGAAAGCAATGAAGAAGGAATATGAAATGACAAATCTTAGATTGA
Y706-SyrianLocal          TGGTGGACTATTTTAAGAAAGCAATGAAGAAGGAATATGAAATGACAAATCTTAGATTGA
Y708-Niva-95              TGGTGGACTATTTTAAGAAAGCAATGAAGAAGGAATATGAAATGACAAATCTTAGATTGA
Y704-Flip92-36L           TGGTGGACTATTTTAAGAAAGCAATGAAGAAGGAATATGAAATGACAAATCTTAGATTGA
Y703-Krapinka             TGGTGGACTATTTTAAGAAAGCAATGAAGAAGGAATATGAAATGACAAATCTTAGATTGA
Y701-Flip96-48L           TGGTGGACTATTTTAAGAAAGCAATGAAGAAGGAATATGAAATGACAAATCTTAGATTGA
Y702-ILL-1552             TGGTGGACTATTTTAAGAAAGCAATGAAGAAGGAATATGAAATGACAAATCTTAGATTGA
Y707-Vekhovskaya-1        TGGTGGACTATTTTAAGAAAGCAATGAAGAAGGAATATGAAATGACAAATCTTAGATTGA
                          ************************************************************

Y705-LebaneseLocal        TGAGATATTTCCTTGGAATTCAAGTAAAGCAAATAAAAGGGGAGATTTTTATCACTAGTA
Y706-SyrianLocal          TGAGATATTTCCTTGGAATTCAAGTAAAGCAAATAAAAGGGGAGATTTTTATCACTAGTA
Y708-Niva-95              TGAGATATTTCCTTGGAATTCAAGTAAAGCAAATAAAAGGGGAGATTTTTATCACTAGTA
Y704-Flip92-36L           TGAGATATTTCCTTGGAATTCAAGTAAAGCAAATAAAAGGGGAGATTTTTATCACTAGTA
Y703-Krapinka             TGAGATATTTCCTTGGAATTCAAGTAAAGCAAATAAAAGGGGAGATTTTTATCACTAGTA
Y701-Flip96-48L           TGAGATATTTCCTTGGAATTCAAGTAAAGCAAATAAAAGGGGAGATTTTTATCACTAGTA
Y702-ILL-1552             TGAGATATTTCCTTGGAATTCAAGTAAAGCAAATAAAAGGGGAGATTTTTATCACTAGTA
Y707-Vekhovskaya-1        TGAGATATTTCCTTGGAATTCAAGTAAAGCAAATAAAAGGGGAGATTTTTATCACTAGTA
                          ************************************************************

Y705-LebaneseLocal        CGTTTTAGATATGTTTAAAAATTCAGAATGGAGAATTGTAAACCAGCGTCATAGAATTGT
Y706-SyrianLocal          CGTTTTAGATATGTTTAAAAATTCAGAATGGAGAATTGTAAACCAGCGTCATAGAATTGT
Y708-Niva-95              CGTTTTAGATATGTTTAAAAATTCAGAATGGAGAATTGTAAACCAGCGTCATAGAATTGT
Y704-Flip92-36L           CGTTTTAGATATGTTTAAAAATTCAGAATGGAGAATTGTAAACCAGCGTCATAGAATTGT
Y703-Krapinka             CGTTTTAGATATGTTTAAAAATTCAGAATGGAGAATTGTAAACCAGCGTCATAGAATTGT
Y701-Flip96-48L           CGTTTTAGATATGTTTAAAAATTCAGAATGGAGAATTGTAAACCAGCGTCATAGAATTGT
Y702-ILL-1552             CGTTTTAGATATGTTTAAAAATTCAGAATGGAGAATTGTAAACCAGCGTCATAGAATTGT
Y707-Vekhovskaya-1        CGTTTTAGATATGTTTAAAAATTCAGAATGGAGAATTGTAAACCAGCGTCATAGAATTGT
                          ************************************************************

Y705-LebaneseLocal        AAACTAGAATGAAGAAGTACGTTTTAAATATGCTTGAAAAATTCAAAATGGATTGTAAAC
Y706-SyrianLocal          AAACTAGAATGAAGAAGTACGTTTTAAATATGCTTGAAAAATTCAAAATGGATTGTAAAC
Y708-Niva-95              AAACTAGAATGAAGAAGTACGTTTTAAATATGCTTGAAAAATTCAAAATGGATTGTAAAC
Y704-Flip92-36L           AAACTAGAATGAAGAAGTACGTTTTAAATATGCTTGAAAAATTCAAAATGGATTGTAAAC
Y703-Krapinka             AAACTAGAATGAAGAAGTACGTTTTAAATATGCTTGAAAAATTCAAAATGGATTGTAAAC
Y701-Flip96-48L           AAACTAGAATGAAGAAGTACGTTTTAAATATGCTTGAAAAATTCAAAATGGATTGTAAAC
Y702-ILL-1552             AAACTAGAATGAAGAAGTACGTTTTAAATATGCTTGAAAAATTCAAAATGGATTGTAAAC
Y707-Vekhovskaya-1        AAACTAGAATGAAGAAGTACGTTTTAAATATGCTTGAAAAATTCAAAATGGATTGTAAAC
                          ************************************************************

Y705-LebaneseLocal        CAACATCGTAGAATTGTAAACTAGAATGGAGAAGTATGTTTTCCCATATGATACTCATTC
Y706-SyrianLocal          CAACATCGTAGAATTGTAAACTAGAATGGAGAAGTATGTTTTCCCATATGATACTCATTC
Y708-Niva-95              CAACATCGTAGAATTGTAAACTAGAATGGAGAAGTATGTTTTCCCATATGATACTCATTC
Y704-Flip92-36L           CAACATCGTAGAATTGTAAACTAGAATGGAGAAGTATGTTTTCCCATATGATACTCATTC
Y703-Krapinka             CAACATCGTAGAATTGTAAACTAGAATGGAGAAGTATGTTTTCCCATATGATACTCATTC
Y701-Flip96-48L           CAACATCGTAGAATTGTAAACTAGAATGGAGAAGTATGTTTTCCCATATGATACTCATTC
Y702-ILL-1552             CAACATCGTAGAATTGTAAACTAGAATGGAGAAGTATGTTTTCCCATATGATACTCATTC
Y707-Vekhovskaya-1        CAACATCGTAGAATTGTAAACTAGAATGGAGAAGTATGTTTTCCCATATGATACTCATTC
                          ************************************************************
                                           SNP-2
Y705-LebaneseLocal        TGATCATAGATAGAAAAAAACATAGGAAATCTAACTTTAAGAGAAGTGACCCTCATCTAA
Y706-SyrianLocal          TGATCATAGATAGAAAAAAACATAGGAAATCTAACTTTAAGAGAAGTGACCCTCATCTAA
Y708-Niva-95              TGATCATAGATAGAAAAAAACATAGGAAATCTAACTTTAAGAGAAGTGACCCTCATCTAA
Y704-Flip92-36L           TGATCATAGATAGAAAAAAACATAGGAAATCTAACTTTAAGAGAAGTGACCCTCATCTAA
Y703-Krapinka             TGATCATAGATAGAAAAAAACATAGGAAATCTAACTTTAAGAGAAGTGACCCTCATCTAA
Y701-Flip96-48L           TGATCATAGATAGAAAAAAACATAGGAAATCTAACTTTAAGAGAAGTGACCCTCATCTAA
Y702-ILL-1552             TGATCATAGATAGAAAAAAGCATAGGAAATCTAACTTTAAGAGAAGTGACCCTCATCTAA
Y707-Vekhovskaya-1        TGATCATAGATAGAAAAAAGCATAGGAAATCTAACTTTAAGAGAAGTGACCCTCATCTAA
                          ******************* ****************************************
```

```
Y705-LebaneseLocal      GGATCACTCTAGGAAGAAGTGAGTAGACCTTATCTAACCTGTTTAAGCAAACCGTCTCGA
Y706-SyrianLocal        GGATCACTCTAGGAAGAAGTGAGTAGACCTTATCTAACCTGTTTAAGCAAACCGTCTCGA
Y708-Niva-95            GGATCACTCTAGGAAGAAGTGAGTAGACCTTATCTAACCTGTTTAAGCAAACCGTCTCGA
Y704-Flip92-36L         GGATCACTCTAGGAAGAAGTGAGTAGACCTTATCTAACCTGTTTAAGCAAACCGTCTCGA
Y703-Krapinka           GGATCACTCTAGGAAGAAGTGAGTAGACCTTATCTAACCTGTTTAAGCAAACCGTCTCGA
Y701-Flip96-48L         GGATCACTCTAGGAAGAAGTGAGTAGACCTTATCTAACCTGTTTAAGCAAACCGTCTCGA
Y702-ILL-1552           GGATCACTCTAGGAAGAAGTGAGTAGACCTTATCTAACCTGTTTAAGCAAACCGTCTCGA
Y707-Vekhovskaya-1      GGATCACTCTAGGAAGAAGTGAGTAGACCTTATCTAACCTGTTTAAGCAAACCGTCTCGA
                        ************************************************************

Y705-LebaneseLocal      AACTAGTCAGAATGGTCCTCCACTATAACCCCTACAAGAGACACCCCTCTCAATCAAGGA
Y706-SyrianLocal        AACTAGTCAGAATGGTCCTCCACTATAACCCCTACAAGAGACACCCCTCTCAATCAAGGA
Y708-Niva-95            AACTAGTCAGAATGGTCCTCCACTATAACCCCTACAAGAGACACCCCTCTCAATCAAGGA
Y704-Flip92-36L         AACTAGTCAGAATGGTCCTCCACTATAACCCCTACAAGAGACACCCCTCTCAATCAAGGA
Y703-Krapinka           AACTAGTCAGAATGGTCCTCCACTATAACCCCTACAAGAGACACCCCTCTCAATCAAGGA
Y701-Flip96-48L         AACTAGTCAGAATGGTCCTCCACTATAACCCCTACAAGAGACACCCCTCTCAATCAAGGA
Y702-ILL-1552           AACTAGTCAGAATGGTCCTCCACTATAACCCCTACAAGAGACACCCCTCTCAATCAAGGA
Y707-Vekhovskaya-1      AACTAGTCAGAATGGTCCTCCACTATAACCCCTACAAGAGACACCCCTCTCAATCAAGGA
                        ************************************************************

Y705-LebaneseLocal      GACACATATAGAAGACCCCAAACTCTACTATCCAAAATAGACAAAATCGACGTACCCCAT
Y706-SyrianLocal        GACACATATAGAAGACCCCAAACTCTACTATCCAAAATAGACAAAATCGACGTACCCCAT
Y708-Niva-95            GACACATATAGAAGACCCCAAACTCTACTATCCAAAATAGACAAAATCGACGTACCCCAT
Y704-Flip92-36L         GACACATATAGAAGACCCCAAACTCTACTATCCAAAATAGACAAAATCGACGTACCCCAT
Y703-Krapinka           GACACATATAGAAGACCCCAAACTCTACTATCCAAAATAGACAAAATCGACGTACCCCAT
Y701-Flip96-48L         GACACATATAGAAGACCCCAAACTCTACTATCCAAAATAGACAAAATCGACGTACCCCAT
Y702-ILL-1552           GACACATATAGAAGACCCCAAACTCTACTATCCAAAATAGACAAAATCGACGTACCCCAT
Y707-Vekhovskaya-1      GACACATATAGAAGACCCCAAACTCTACTATCCAAAATAGACAAAATCGACGTACCCCAT
                        ************************************************************

Y705-LebaneseLocal      GTCTAGCGGATAAAATATCACACCAAATAAACACTTGAAA
Y706-SyrianLocal        GTCTAGCGGATAAAATATCACACCAAATAAACACTTGAAA
Y708-Niva-95            GTCTAGCGGATAAAATATCACACCAAATAAACACTTGAAA
Y704-Flip92-36L         GTCTAGCGGATAAAATATCACACCAAATAAACACTTGAAA
Y703-Krapinka           GTCTAGCGGATAAAATATCACACCAAATAAACACTTGAAA
Y701-Flip96-48L         GTCTAGCGGATAAAATATCACACCAAATAAACACTTGAAA
Y702-ILL-1552           GTCTAGCGGATAAAATATCACACCAAATAAACACTTGAAA
Y707-Vekhovskaya-1      GTCTAGCGGATAAAATATCACACCAAATAAACACTTGAAA
                        ****************************************
```
